# Multiple Probiotic Lactic Acid Bacteria with Marked Cholesterol-Lowering Effects Were Discovered in Equine Colostrum

**DOI:** 10.3390/ijms27177816

**Published:** 2026-08-31

**Authors:** Xuelian Liu, Kuerdan Hudayibieergen, Baheban Yeerjiang, Tabusi Manaer, Xinhua Nabi

**Affiliations:** 1 School of Pharmacy, Xinjiang Medical University, Urumchi 830017, China; 17690609892@163.com (X.L.); kuerdan_h@163.com (K.H.); 18999742219@163.com (B.Y.); tbs2233@163.com (T.M.); 2 Xinjiang Key Laboratory of Natural Medicines Active Components and Drug Release Technology, Xinjiang Medical University, Urumchi 830017, China; 3 Engineering Research Center of Xinjiang and Central Asian Medicine Resources, Ministry of Education, Xinjiang Medical University, Urumchi 830017, China

**Keywords:** equine colostrum, probiotic screening, hypocholesterolemic activity, gut microbiota, bile acid metabolism, short-chain fatty acids

## Abstract

Xinjiang ranks among the key domestication origins of horses. Local nomadic ethnic groups have a long-standing tradition of consuming equine dairy products, which endows mare-derived dairy matrices with abundant, distinctive and scarce probiotic resources. Equine colostrum is rich in immunoglobulins and growth factors; nevertheless, its pharmacological activities and micro-ecological composition remain poorly characterized. This study aimed to isolate and screen native strains with cholesterol-lowering potential from Xinjiang equine colostrum and conduct a preliminary investigation into their underlying mechanisms. Following isolation and taxonomic identification, one strain of *Lactobacillus helveticus*, four strains of *Lactiplantibacillus plantarum*, and two yeast strains including *Pichia fermentans* and *Pichia membranifaciens* were obtained. All the aforementioned isolates are indigenous microorganisms naturally inhabiting mare colostrum. In vitro assays demonstrated bile salt hydrolase activity and cholesterol clearance rates ranging from 45.07% to 87.14%, with composite probiotic formulations exhibiting superior efficacy. In vivo results demonstrated that the combination formulation significantly reduced serum levels of total cholesterol, triglycerides, and low-density lipoprotein cholesterol (*p* < 0.05), alleviated hepatic lipid deposition, and regulated gut microbiota composition—notably by enhancing the abundance of beneficial bacteria. It also increased fecal short-chain fatty acids and decreased bile acid levels. These findings demonstrate that the native strains isolated from mare colostrum possess desirable probiotic properties and lipid-lowering bioactivities. Their unique source and natural combinatorial characteristics provide candidate-strain resources for the development of novel functional foods and nutritional supplements.

## 1. Introduction

Atherosclerosis constitutes the pathological basis for numerous cardiovascular diseases, and hyperlipidemia acts as a major pathogenic factor for cardiovascular disorders. Statins, the representative drugs for atherosclerosis and hyperlipidemia therapy, are microbial-derived compounds [1,2]. Clinically validated as first-line agents, statins reduce morbidity and mortality in patients at high risk of atherosclerotic cardiovascular disease (ASCVD), exert broad lipid-modulating effects, and confer cardioprotective benefits.

As microecological regulators, probiotics have attracted substantial attention owing to their promising potential in blood-lipid management. Our previous studies have demonstrated that cholesterol-lowering strains screened from diverse fermented dairy products ameliorate insulin resistance both in vivo and in vitro, and such effects are closely associated with the regulation of cholesterol metabolism [3]. Cumulative domestic and international evidence has confirmed that lactic acid bacteria and certain yeasts exert lipid-lowering effects by remodeling gut microbiota composition and producing bioactive metabolites [4,5]. Nevertheless, existing investigations have largely focused on probiotics isolated from conventional fermented dairy products such as cow’s milk, goat’s milk and koumiss, while microbial exploitation and functional research on the special biological resource of mare colostrum remain insufficient.

As one of the earliest regions for horse domestication, Xinjiang has a long-standing local custom of consuming fermented and raw mare milk. Mare milk, especially mare colostrum, has been traditionally applied for physical conditioning and immune-function improvement; however, the key bioactive components and underlying mechanisms responsible for such effects have not been scientifically elucidated. Equine colostrum refers to the natural milk secreted by mares within the first few days postpartum without any fermentation processing. It is rich in immunoglobulins, lactoferrin, lysozyme and multiple growth factors. Its distinctive nutritional profile and microecological environment render it a unique germplasm resource for screening functional strains with special physiological activities. Current domestic and international studies on probiotic-mediated lipid-lowering effects mostly employ ordinary cow’s milk, goat’s milk or mature fermented koumiss as starting materials, and few efforts have been devoted to exploring native microorganisms from equine colostrum [6,7,8].

More importantly, lactic acid bacteria and yeasts naturally residing in mare colostrum are not artificially combined but represent a naturally symbiotic system shaped by long-term co-evolution. During fermentation, yeasts supply lactic acid bacteria with diverse nutritional factors including amino acids, vitamins and pyruvate, and secrete proteases and lipases to decompose substrates and generate growth-promoting substances. In return, metabolic products from lactic acid bacteria serve as energy sources for yeasts, forming metabolic complementarity between the two groups. Such properties may endow them with superior functional synergy compared to conventionally artificially compounded probiotics [9].

Accordingly, equine colostrum from Xinjiang was selected as the microbial source in the present study. On the one hand, Xinjiang possesses abundant horse resources, and local residents have maintained a long-term tradition of consuming mare milk and fermented mare-milk products. On the other hand, the unique microecology of mare colostrum holds great promise for screening novel and high-efficiency cholesterol-lowering strains. Through systematic in vitro and in vivo experiments, this study clarified the cholesterol-lowering efficacy and potential mechanisms of probiotics derived from equine colostrum. It provides a novel microecological modulation strategy for the adjuvant treatment of hyperlipidemia and further promotes the research and application of probiotics in the field of blood-lipid management.

## 2. Results

### 2.1. Isolation and Identification of Bacterial Strains Based on Morphological, Physiological–Biochemical, and Molecular Characteristics

#### 2.1.1. Strains Isolation Results

**Figure 1:**
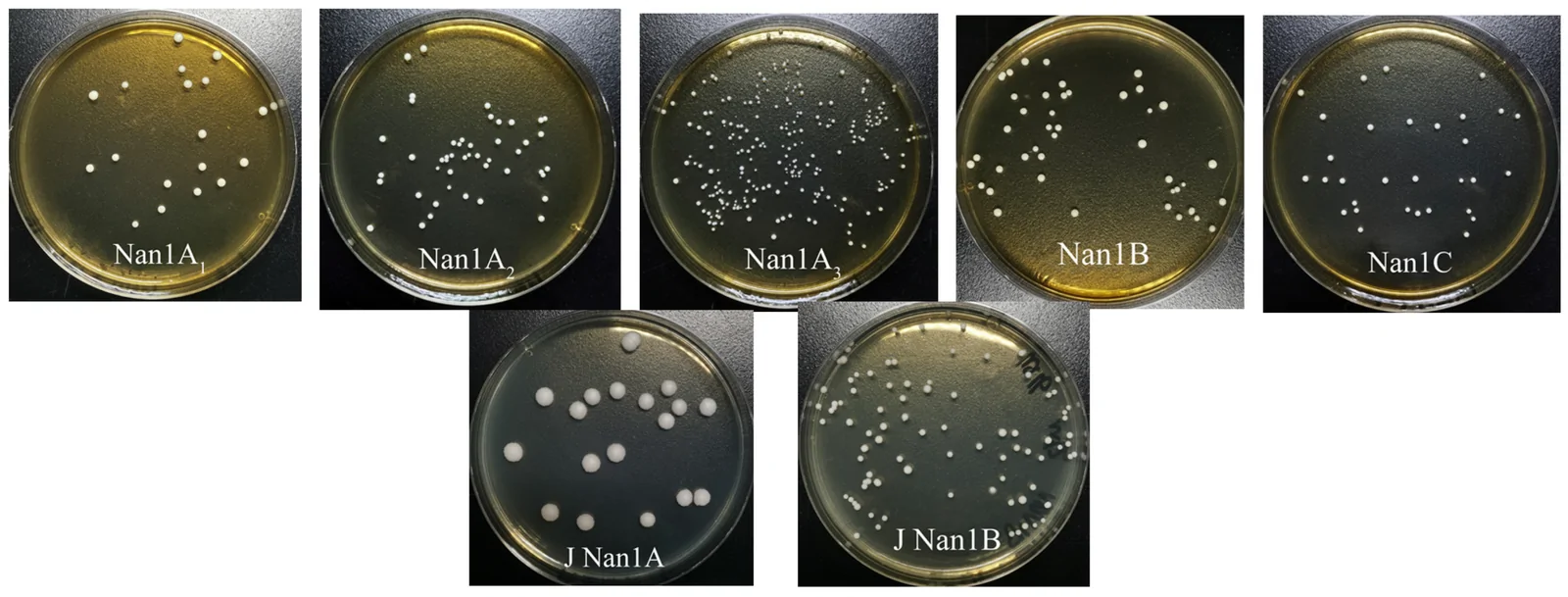
Colony Characteristics of Lactic Acid Bacteria and Yeast.

Based on colony morphology, microscopic observation, Gram staining, and catalase testing (3% H_2_O_2_), five strains were identified as lactic acid bacteria (LAB) and designated as Nan1A_1_, Nan1A_2_, Nan1A_3_, Nan1B, and Nan1C; two strains were identified as yeasts and designated as JNan1A and JNan1B. Their morphological characteristics are shown in Figure 1 (Supplementary Table S1). All five LAB strains formed convex colonies with moist, smooth, and glossy surfaces and with neat margins. In contrast, both yeast strains produced flat, viscous colonies that had irregular and rough margins with a matte appearance.

#### 2.1.2. Results of Physiological and Biochemical Identification

All five LAB strains were rod-shaped, tested negative for oxidase activity and indole production, and did not liquefy gelatin. Among them, Nan1A_2_ and Nan1A_3_ could grow at 45 °C, whereas none grew in medium containing 6.5% NaCl. Carbon source utilization profiles were analyzed using the API 50 CH system (Supplementary Table S2). Yeast cells exhibited ellipsoidal morphology and were capable of growth on malt medium. Both strains formed pseudohyphae on Dalmau cornmeal agar, with JNan1A showing growth at 37 °C. Sugar fermentation tests (Supplementary Table S3) revealed that neither strain fermented galactose, sucrose, maltose, lactose, or raffinose; only JNan1A fermented glucose. Carbon source assimilation assays (Supplementary Table S4) demonstrated that both strains assimilated glucose, ethanol, lactic acid, and succinate. Among these, JNan1A assimilated citrate and was weakly positive for D-xylose, whereas JNan1B was negative for both. In nitrogen source assimilation tests (Supplementary Table S5), neither strain assimilated nitrate, creatine, cadaverine hydrochloride, or lysine.

#### 2.1.3. Gene Sequence Identification Results

**Figure 2:**
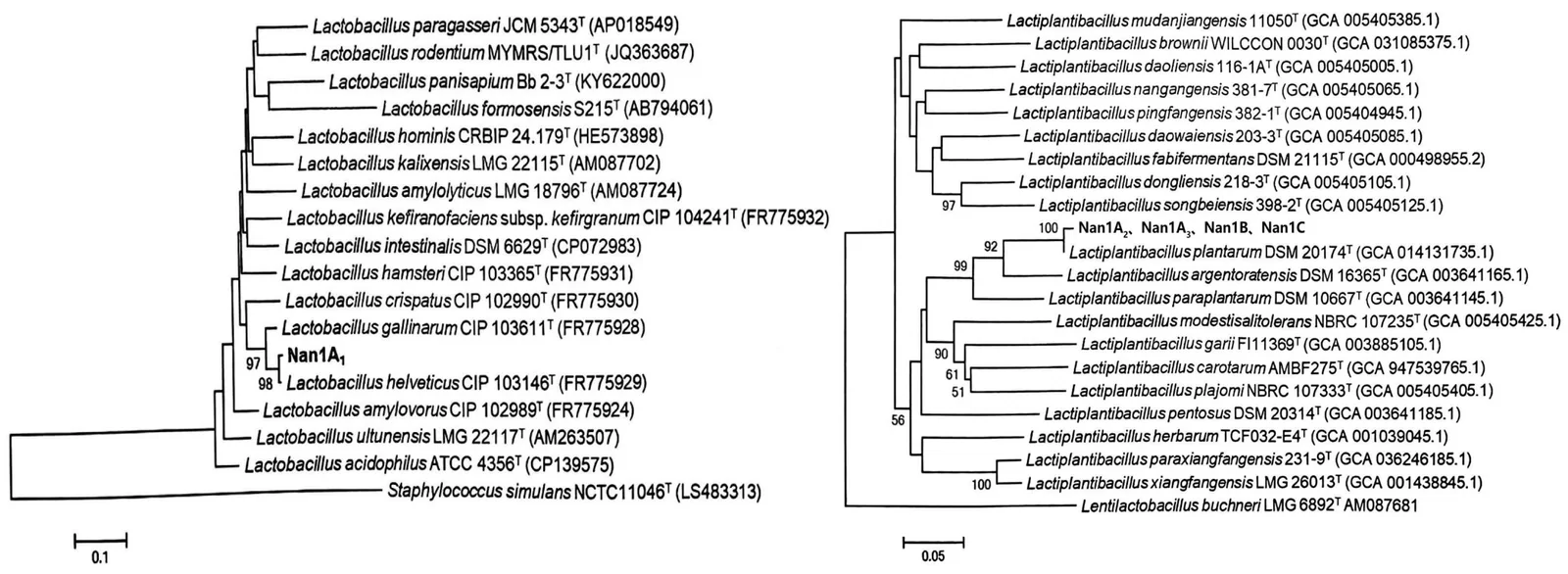
Phylogenetic tree of lactic acid bacteria from equine colostrum based on 16S rRNA gene sequences.

The five lactic acid bacteria strains were identified based on polyphasic taxonomy, following the principles outlined in Bergey’s Manual of Systematic Bacteriology and the International Journal of Systematic and Evolutionary Microbiology. This involved analysis of 16S rRNA and pheS gene sequences combined with relevant phenotypic characteristics. As shown in Figure 2, strain Nan1A_1_ was identified as *Lactobacillus helveticus*, while the remaining four strains (Nan1A_2_, Nan1A_3_, Nan1B, and Nan1C) were identified as *Lactiplantibacillus plantarum*. The yeast strains JNan1A and JNan1B were identified through a comprehensive analysis of their cytomorphology, culture characteristics, physiological and biochemical properties, and D1/D2 domain sequences of the 26S rRNA gene, with reference to the same standard resources. Consequently, strain JNan1A was identified as *Pichia fermentans*, and strain JNan1B as *Pichia membranifaciens*.

### 2.2. Probiotic Properties (Growth Curve, Bile Salt Tolerance, Gastric Juice Tolerance, Auto-Aggregation, Surface Hydrophobicity, and Caco-2 Cell Adhesion) and Antibiotic Susceptibility of the Isolated Strain

#### 2.2.1. Growth Curve

**Figure 3:**
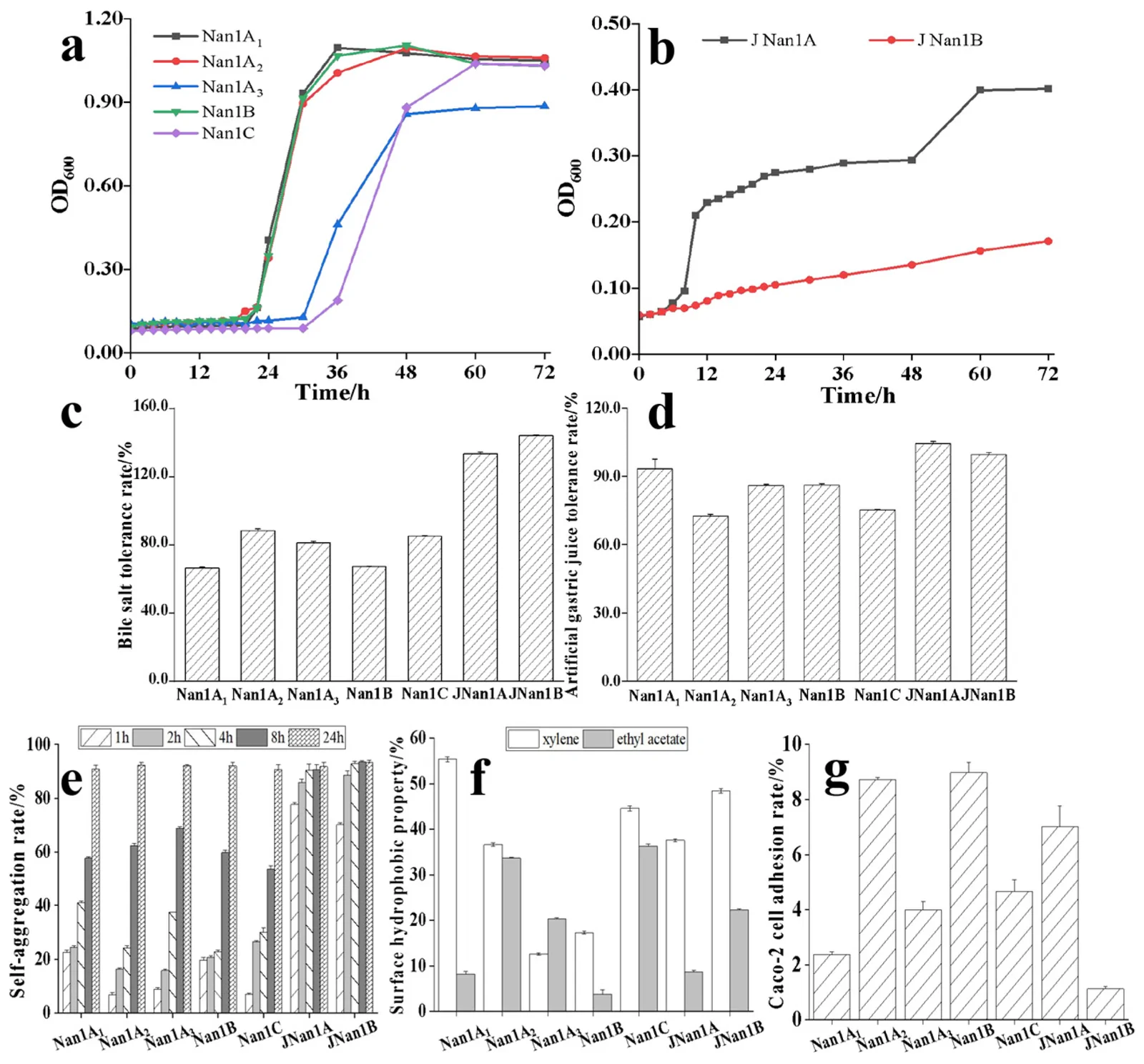
Probiotic Property Evaluation. (**a**): Lactic acid bacteria growth curve; (**b**): Yeast growth curve; (**c**): Bile salt tolerance rate; (**d**): Tolerance to simulated gastric fluid; (**e**): Auto aggregation Capacity; (**f**): Surface hydrophobicity rate; (**g**): Caco-2 cell adhesion rate.

As shown in Figure 3a, all five LAB strains grew robustly in MRS broth, albeit with varying onset times for the logarithmic growth phase. Nan1A_2_ entered this phase earliest (20 h), followed by Nan1A_1_ and Nan1B (22 h). Nan1A_3_ and Nan1C entered later, at 30 h and 36 h, respectively. Following 48 h of cultivation, all strains had reached the stationary phase, indicating that growth had plateaued.

As shown in Figure 3b, the growth patterns of the yeast strains also differed significantly. JNan1A entered the logarithmic phase after 8 h of cultivation and exhibited a secondary growth phase at 48 h, whereas JNan1B demonstrated sustained slow growth throughout the 72 h cultivation period. This observation aligns with findings reported by Zhao et al. [10], indicating that all strains undergo a slow-growth phase before entering the logarithmic phase and ultimately reaching the stationary phase.

#### 2.2.2. Bile Salt Tolerance

As shown in Figure 3c, after 3 h of anaerobic cultivation under high bile salt conditions (3.0 g/L), the survival rates of the five LAB strains ranged from 66.47% to 88.39% relative to the control. Among these, Nan1A_2_ exhibited the highest survival rate, whereas Nan1A_1_ showed the lowest. Under identical conditions, the yeast strains JNan1A and JNan1B showed survival rates of 133.52% and 144.06%, respectively, indicating not only tolerance but potential growth stimulation. These results demonstrate that all tested strains possess notable tolerance to elevated bile salt concentrations, with significant inter-strain variation. This aligns with a fundamental requirement for probiotic function within the physiological bile salt concentration range of the human small intestine (0.3–3.0 g/L).

#### 2.2.3. Tolerance to Simulated Gastric Fluid

As shown in Figure 3d, after a 3 h exposure to simulated gastric fluid (pH 2.0), the survival rates of the five LAB strains ranged from 72.55% to 93.30%, with Nan1A_1_ exhibiting the highest rate and Nan1A_2_ the lowest. Under identical conditions, the yeast strains JNan1A and JNan1B showed survival rates of 104.44% and 99.59%, respectively. These results demonstrate that all tested strains exhibit notable tolerance to simulated gastric conditions. This finding is consistent with the study by Bhushan et al. [11], which indicates that gastric fluid tolerance can vary among different strains of the same species, and that survival over a 3 h period supports their ability to withstand gastric digestion.

#### 2.2.4. Autoaggregation Capacity

Autoaggregation, the ability of microbial cells to adhere to each other, can increase local cell density in the gut, thereby promoting adhesion to the intestinal epithelium and enhancing colonization [12]. As shown in Figure 3e, all tested strains exhibited strong autoaggregation capacity. The aggregation rates exceeded 10%, 20%, and 90% after 2, 4, and 24 h of incubation, respectively. Notably, the yeast strains displayed the most rapid autoaggregation, achieving rates greater than 70% within 1 h (JNan1A: 77.73% ± 0.67%; JNan1B: 70.16% ± 0.60%). This trend of increasing autoaggregation over time is consistent with the findings of Song et al. [13], in that the auto-aggregation of the strains increased with prolonged incubation time; after 24 h, the autoaggregation rate exceeded 90% and the supernatant was clear, indicating that the strains possess the potential to promote intestinal colonization through aggregation.

#### 2.2.5. Surface Hydrophobicity

The surface hydrophobicity of the bacterial strains was assessed using two organic solvents, xylene and ethyl acetate (Figure 3f). Hydrophobicity toward xylene ranged from 12.56% to 55.36%, with Nan1A_1_ showing the highest value and Nan1B the lowest. For ethyl acetate, the values ranged from 8.16% to 36.30%, where Nan1C exhibited the highest hydrophobicity and Nan1A_1_ the lowest. With the exception of Nan1A_3_, all strains displayed greater hydrophobicity toward xylene than toward ethyl acetate.

#### 2.2.6. Adhesion Capacity to Caco-2 Cells

The synergistic effect of autoaggregation and surface hydrophobicity mediates bacterial adhesion to intestinal epithelial cells. As shown in Figure 3g, all tested strains adhered to Caco-2 cell monolayers, with adhesion rates ranging from 1.13% to 8.98%. Among them, strain Nan1B showed the highest adhesion rate, whereas yeast strain JNan1B showed the lowest. Notably, all strains exhibited adhesion rates above 1%, supporting their potential for intestinal colonization and probiotic function.

#### 2.2.7. Antibiotic Susceptibility Test Results

**Figure 4:**
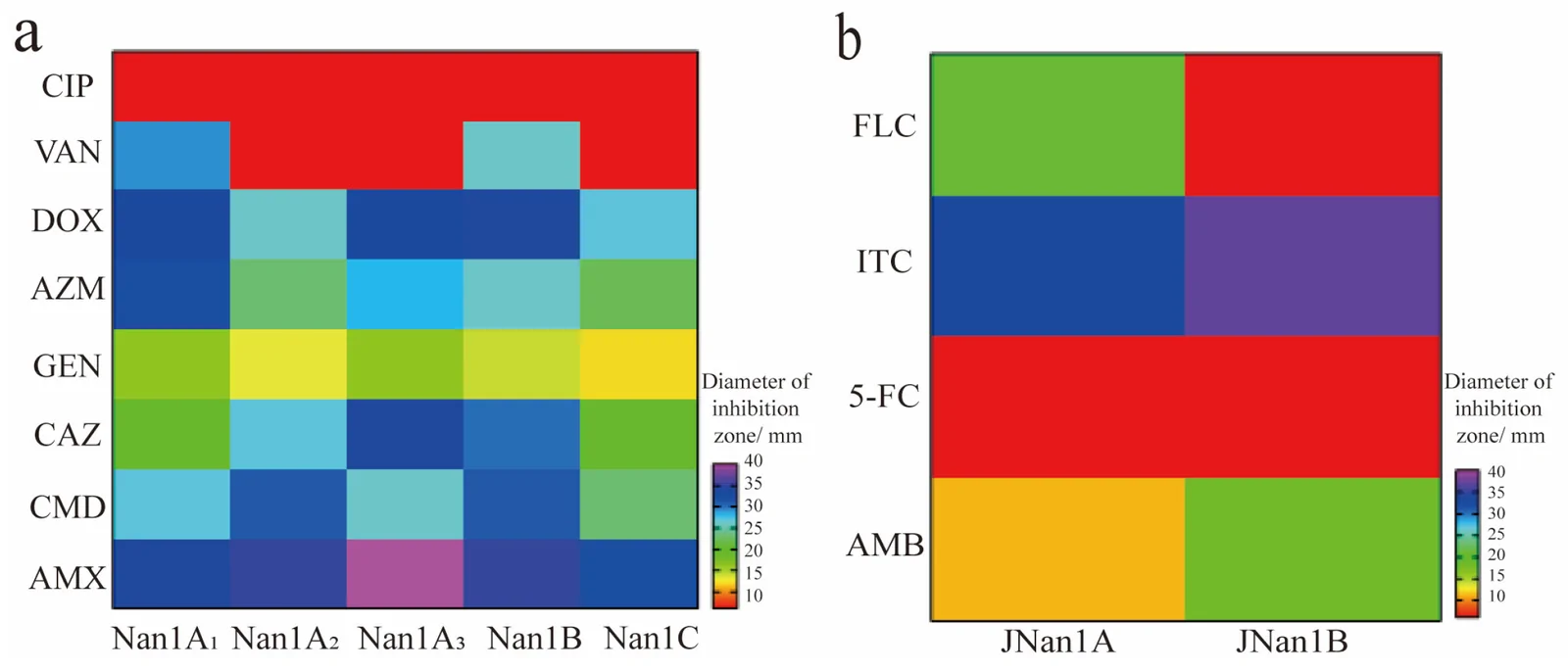
Heatmap Analysis of Antibiotic Resistance. (**a**) Heatmap analysis of antibiotic resistance of lactic acid bacteria; (**b**) Heatmap analysis of antifungal drug resistance of yeasts.

This study evaluated the antimicrobial susceptibility of LAB to eight clinically relevant antibiotics and assessed yeast sensitivity to four antifungal agents. As shown in Figure 4a, the LAB strains were susceptible to amoxicillin (AMX), cefamandole (MAN), ceftazidime (CAZ), azithromycin (AZM), and doxycycline (DOX), and exhibited intermediate susceptibility to gentamicin (GEN)—a finding consistent with prior reports [14]. Resistance was observed against ciprofloxacin (CIP) and vancomycin (VAN), with the latter showing strain-specific variation. Among the antibiotic resistance phenotypes in LAB, resistance to protein synthesis inhibitors (87.5% of strains) and cell wall synthesis inhibitors (62.5%) was predominant [15], corroborating the established intrinsic resistance to quinolones in *Lactobacillus* species [16]. The antifungal susceptibility results for yeasts (Figure 4b) demonstrated sensitivity to itraconazole (ITC), but resistance to 5-fluorocytosine (5-FC) and reduced susceptibility to fluconazole (FLU).

### 2.3. In Vitro and in Vivo Cholesterol-Lowering Effects of the Isolated Strains

#### 2.3.1. In Vitro Cholesterol-Lowering Effects

##### Bile Salt Hydrolase Activity Assay

**Figure 5:**
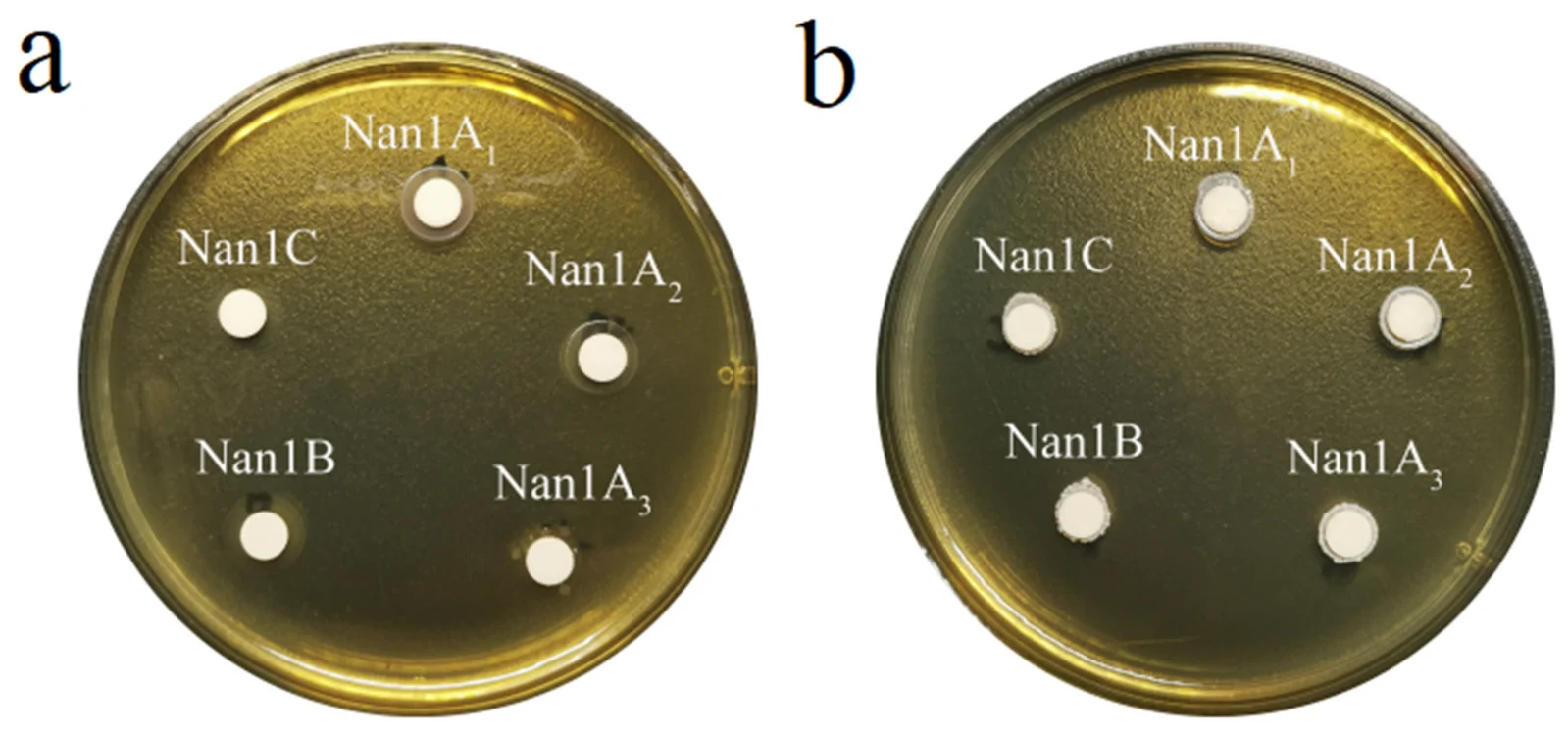
Qualitative assay of BSH activity. (**a**): MRS agar medium without bile salts; (**b**): MRS agar medium with bile salts.

Lactic acid bacteria expressing bile salt hydrolase (BSH) activity deconjugate bile salts in medium supplemented with bile salt substrates. The resultant free bile salts can bind cholesterol to form insoluble complexes [17]. Under acidic conditions, free bile salts precipitate with calcium ions (Ca^2+^) as white crystalline aggregates [18]. As shown in Figure 5b, opaque precipitation halos surrounded the filter disks for two strains, while granular white colonies formed around the disks of the remaining three strains. These observations indicate BSH activity in all five strains. Strain Nan1A_1_ demonstrated the largest precipitation zone diameter, suggesting it possesses the superior BSH activity among them.

##### Cholesterol Degradation Rate

Total cholesterol was quantified using the cholesterol oxidase method, wherein cholesterol is enzymatically converted into a red quinone derivative through the combined action of cholesterol esterase, cholesterol oxidase, and peroxidase. Furthermore, studies indicate that the presence of bile salts facilitates enhanced cholesterol assimilation by lactic acid bacteria [19]. As shown in Table 1, the cholesterol clearance rates ranged from 45.07% to 87.14% for individual strains, low-dose probiotic consortia, and high-dose probiotic consortia. Among the single strains, Nan1C exhibited the highest cholesterol clearance rate, while Nan1A_1_ showed the lowest. The high-dose probiotic consortium achieved a significantly higher clearance rate (81.71% ± 1.06%) than the low-dose probiotic consortium (50.52% ± 0.79%).

#### 2.3.2. In Vivo Hypocholesterolemic Efficacy

##### Effects of a Composite Probiotic on Body Weight and Blood Biochemical Parameters in Rats

**Figure 6:**
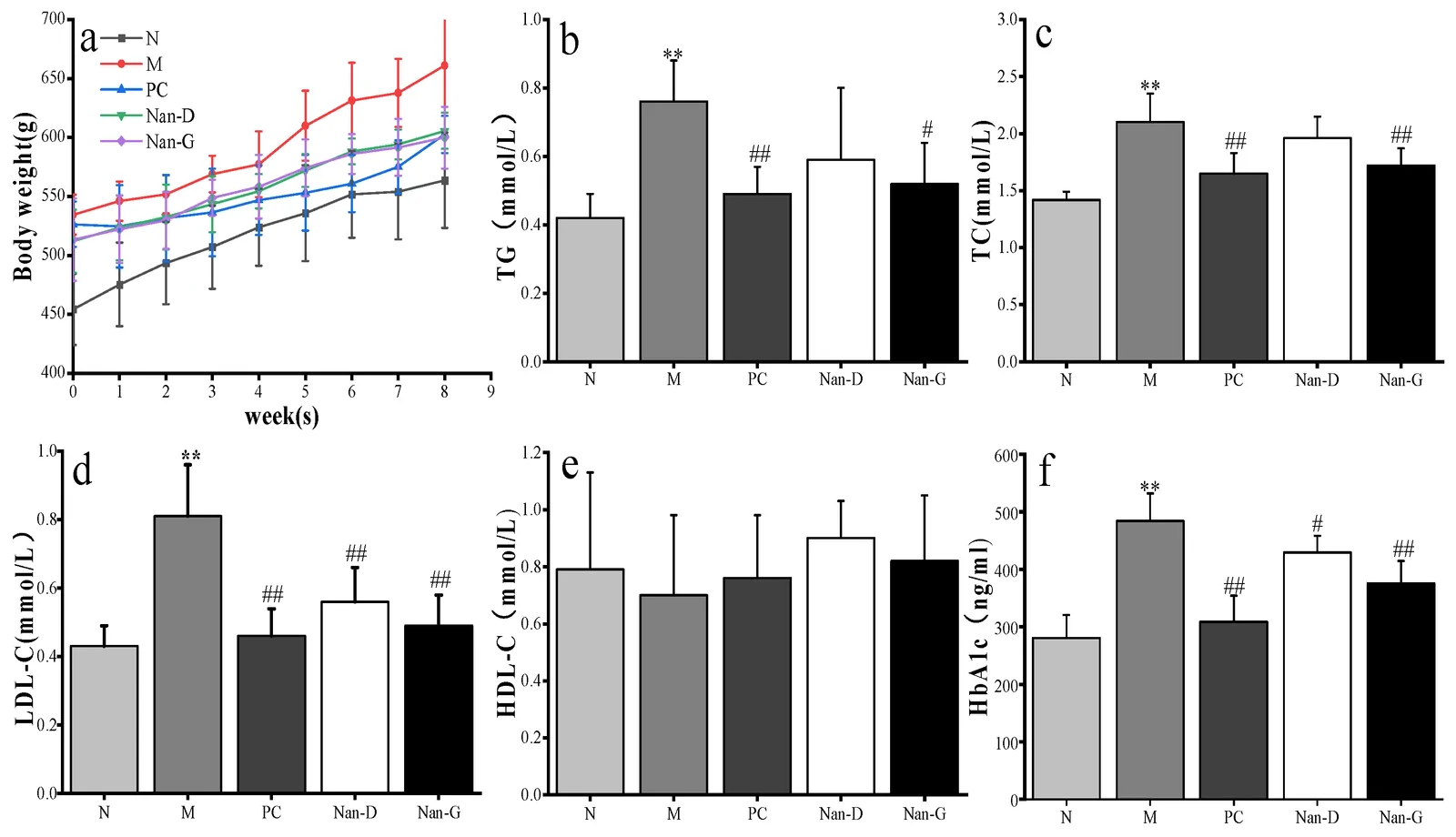
Effects of a Composite Probiotic on Body Weight, Blood Lipids, and Blood Glucose in Hyperlipidemic Rats. (**a**): Body Weight; (**b**): TG; (**c**): TC; (**d**): LDL-C; (**e**): HDL-C; (**f**): HbAlc (n = 8). Data are presented as mean ± SD values. ** *p* <0.01 vs. N, ^#^ *p* < 0.05, ^##^ *p* < 0.01 vs. M.

Observations revealed that rats in the N group exhibited active behavior, steady weight gain, glossy coat condition, and normal food and water intake. Rats in the M group showed reduced activity levels alongside rapid weight gain and increased food consumption. Following intervention, weight gain slowed and activity levels increased compared to the model group. As shown in Figure 6a, rats in the M group exhibited significantly higher body weights than those in the N group (*p* < 0.01). Following intervention, both the PC group and the composite probiotic group exhibited slowed weight gain, indicating that the composite probiotic derived from Equine Colostrum effectively suppressed excessive weight gain induced by an HFD in rats.

The effects of the 8-week intervention on serum lipid profiles are shown in Figure 6b–e. Compared to Group N, Group M exhibited significantly elevated levels of serum TC, TG, and LDL-C (*p* < 0.01). Relative to Group M, both the PC and Nan-G groups showed significant reductions in TC, TG, and LDL-C levels (*p* < 0.05), whereas the Nan-D group displayed a significant decrease only in LDL-C (*p* < 0.01). Additionally, HbA1c levels were significantly higher in Group M than in Group N (*p* < 0.01), while HbA1c levels in both the PC group and the composite probiotic intervention groups were significantly lower compared to Group M (*p* < 0.05),as shown in Figure 6f. These results indicate that the composite probiotics derived from Equine Colostrum effectively ameliorated glucose and lipid metabolic disorders in hyperlipidemic rats, with the Nan-G exhibiting more pronounced regulatory effects. These findings align with the report by Teng et al. [2] on the hypolipidemic activity of *Lactiplantibacillus plantarum* LP104.

##### Effects of Composite Probiotics on Liver Pathology

**Figure 7:**
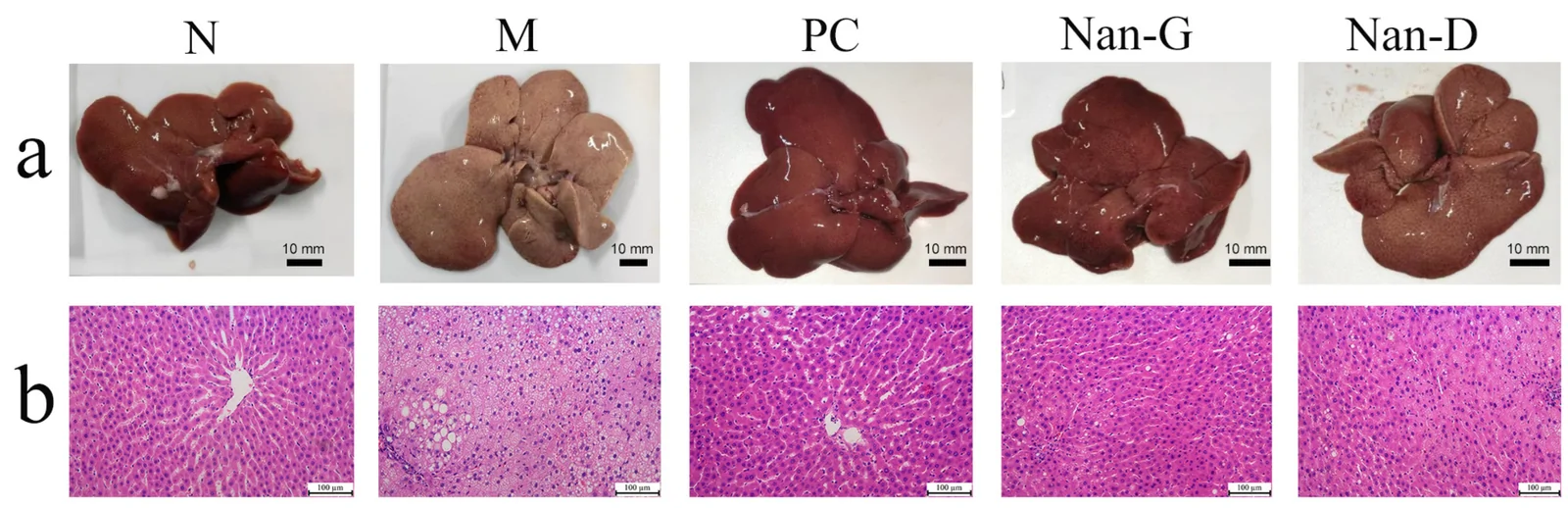
Effects of a Composite Probiotic on the Liver of Hyperlipidemic Rats. (**a**): Macroscopic morphology of the liver; (**b**): HE-stained liver section (200×).

Macroscopic examination of the liver (Figure 7a) revealed that livers from HFD-fed rats in Group M were enlarged and displayed a pale brown discoloration with scattered, lipid-like patches on the surface, accompanied by a greasy and soft texture indicative of substantial lipid deposition, compared to Group N. Following intervention, livers in both the Group PC and composite probiotic groups exhibited a uniform reddish-brown color with smooth surfaces. The lipid-like patches were markedly reduced, resulting in a morphological appearance closely resembling that of Group N.

Histopathological evaluation by hematoxylin and eosin (H&E) staining (Figure 7b) revealed extensive hepatocellular steatosis in Group M. Affected hepatocytes predominantly exhibited microvesicular fat deposition, with most displaying a foamy morphology indicative of severe cellular injury. Comparative post-intervention analysis demonstrated that both the PC and Nan-G groups maintained well-defined hepatic lobular architecture, accompanied by a significant attenuation of steatosis and reduced lipid vacuole formation. In Group Nan-D, partial hepatocellular steatosis persisted, characterized by a mixed pattern of macro- and microvesicular lipid vacuolation, wherein a subset of hepatocytes retained a foamy appearance. These results indicate that the HFD successfully induced lipid metabolism disorders in rat livers, leading to hepatic steatosis and suggesting liver injury. The probiotic complex effectively attenuated HFD-induced hepatic fat accumulation, thereby improving the pathological condition of the liver.

### 2.4. Mechanism of Composite Probiotics in Regulating Blood Lipids

#### 2.4.1. Effects of Composite Probiotics on the Gut Microbiota of Rats

##### Alterations in Gut Microbiota Diversity

**Figure 8:**
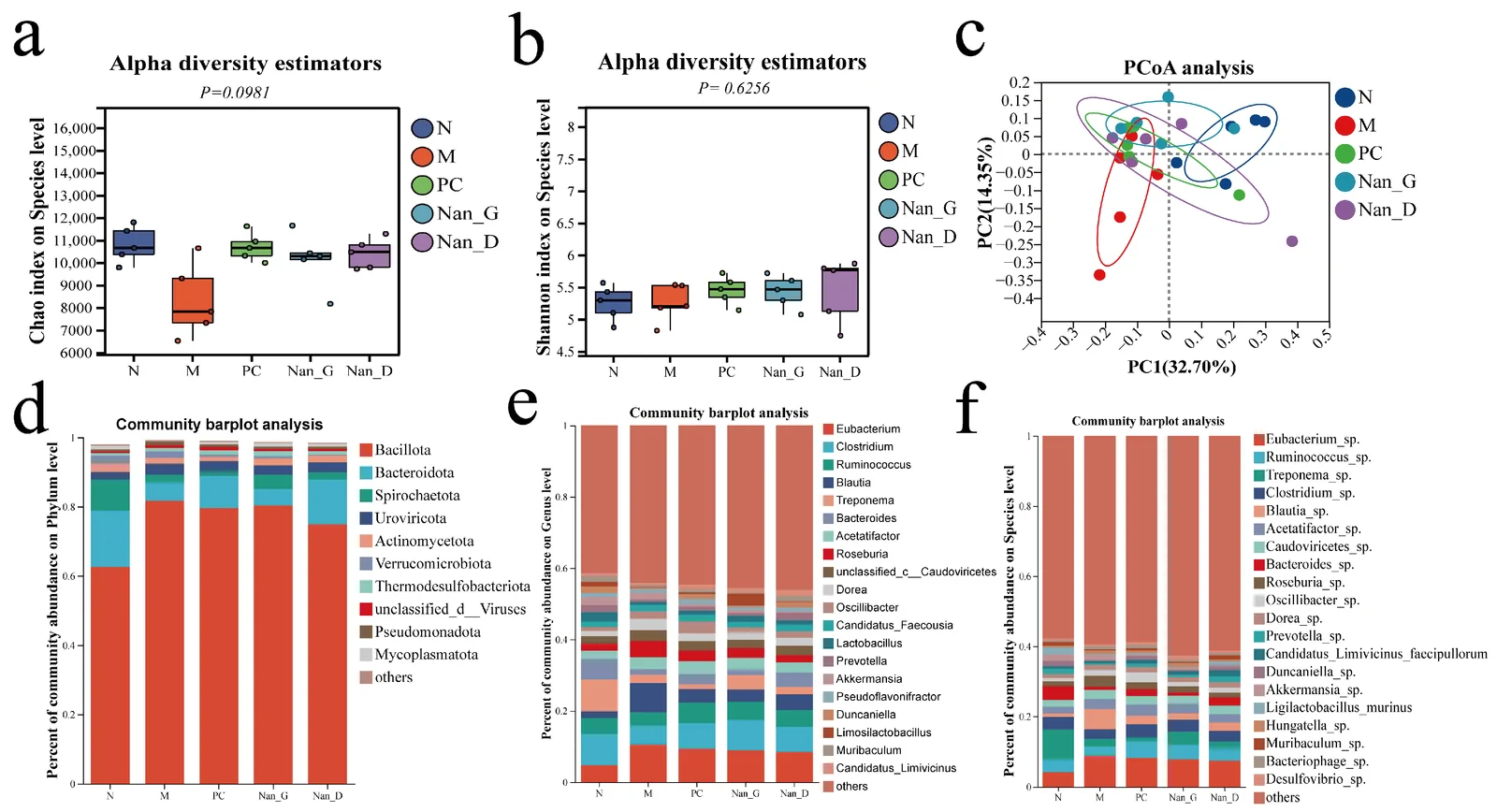
Effects of composite probiotics on gut microbiota diversity and abundance in hyperlipidemic rats. (**a**): Chao index; (**b**): Shannon index; (**c**): PCoA analysis; (**d**): Species abundance at phylum level; (**e**): Species abundance at genus level; (**f**): Species abundance at species level.

This study assessed gut microbiota α-diversity using the Chao index (reflecting species richness) and the Shannon index (reflecting both richness and evenness). As shown in Figure 8a, compared with Group N, Group M exhibited a lower Chao index with greater dispersion. Post-intervention, species richness increased in all treated groups, though intergroup differences were not statistically significant (*p* = 0.098). Regarding the Shannon index (Figure 8b), Group M was significantly lower than Group N. After intervention, all groups showed an increasing trend, but again without statistical significance (*p* = 0.626). PCoA based on the Bray–Curtis distance matrix (Figure 8c) revealed distinct separation between Groups N and M, indicating that the HFD significantly altered gut microbiota structure. Meanwhile, the distributions of all intervention groups shifted toward Group N, suggesting that the interventions ameliorated the gut dysbiosis induced by the HFD.

##### Changes in Gut Microbiota Species Composition

At the phylum level (Figure 8d), the dominant bacterial phyla across all groups were Bacillota, Bacteroidota, and Spirochaetota. Compared to Group N, the Bacillota/Bacteroidota (F/B) ratio was elevated in Group M, whereas this ratio decreased in all intervention groups. At the genus level (Figure 8e), the predominant genera included *Eubacterium*, *Clostridium*, and *Ruminococcus*. Group M exhibited a significant increase in the abundance of *Blautia* and *Roseburia*. In contrast, the intervention groups showed marked increases in beneficial genera such as *Clostridium*, *Ruminococcus*, *Bacteroides*, *Oscillibacter*, *Prevotella*, *Lactobacillus*, and *Limosilactobacillus*. Species-level analysis (Figure 8f) further confirmed that the abundance of beneficial bacteria, including *Ruminococcus*_sp., *Bacteroides*_sp., *Oscillibacter*_sp., and *Prevotella*_sp., was significantly enhanced following intervention. In summary, the composite probiotic intervention increased the abundance of beneficial intestinal bacteria and ameliorated the gut microbiota dysbiosis induced by the HFD.

##### Effects of Composite Probiotics on Microbial Composition

**Figure 9:**
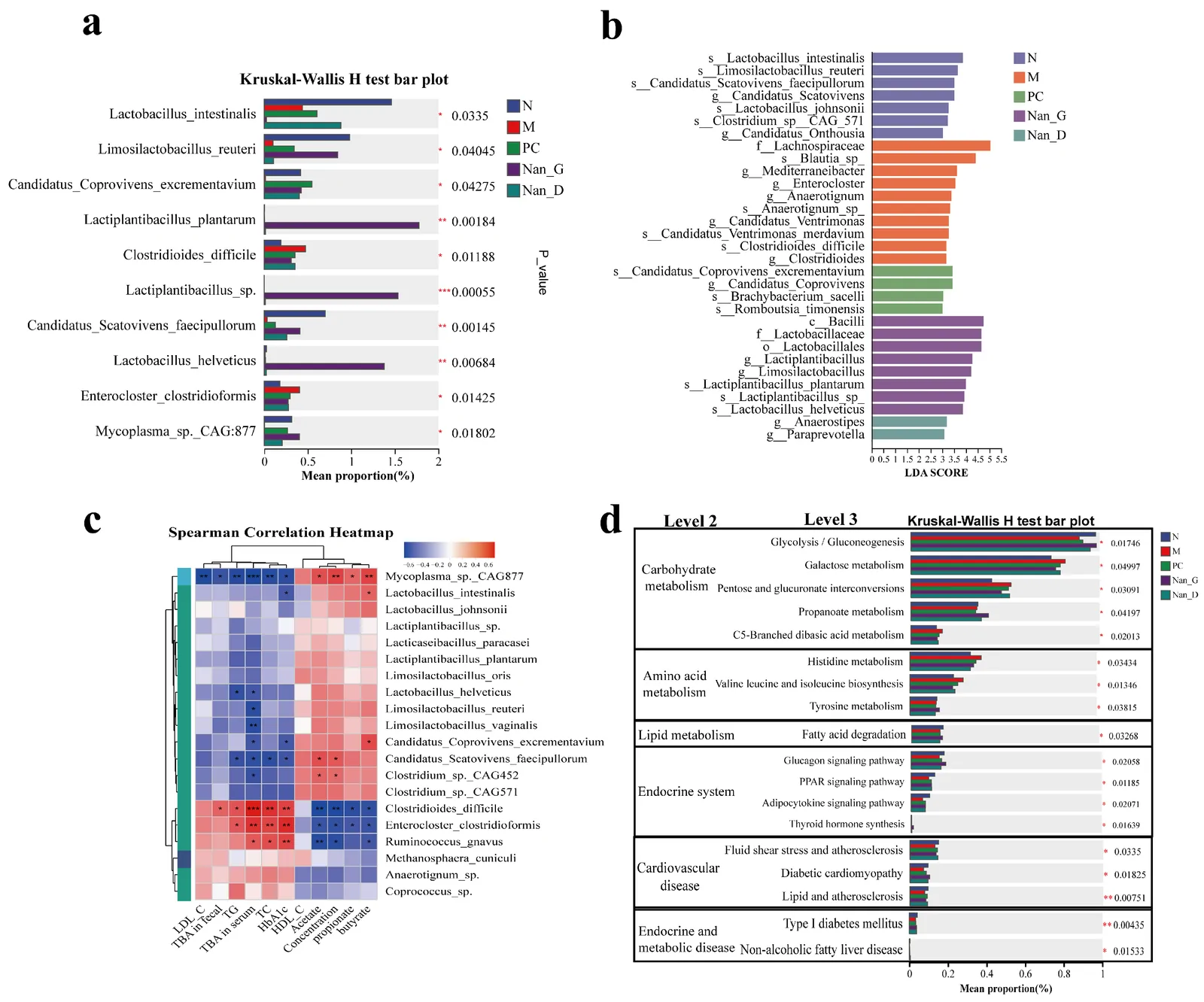
Effects of Composite Probiotics on Gut Microbiota Differences and Function in Hyperlipidemic Rats. (**a**): Species difference test diagram; (**b**): LDA diagram; (**c**): Heatmap of correlations between key gut microbial species and lipid metabolism parameters; (**d**): Functional metabolic difference diagram of gut microbiota. *** *p* < 0.001, ** *p* < 0.01, * *p* < 0.05. These statistical comparisons highlight the significant differences between the groups in the study.

To assess intergroup differences in gut microbiota composition, a species-level differential abundance analysis was performed. The results (Figure 9a) revealed significant intergroup differences in the relative abundance of *Lactiplantibacillus*_sp., *Lactiplantibacillus plantarum*, *Lactobacillus helveticus*, *Lactobacillus intestinalis*, and *Limosilactobacillus reuteri*.

Distinct microbial biomarkers enriched across groups were further identified by LEfSe analysis (LDA score > 3; Figure 9b). Group N was characterized by significant enrichment of *Lactobacillus intestinalis*, *Limosilactobacillus reuteri*, and *Candidatus* Scatovivens faecipullorum. In contrast, Group M was markedly enriched with f__Lachnospiraceae, *Blautia*_sp., and *Mediterraneibacter*. Distinct enrichment patterns were also observed in the intervention groups: the PC group was primarily enriched with *Candidatus* Coprovivens; the Nan-G group showed significant enrichment of *g__Limosilactobacillus* and *g__Lactiplantibacillus*; and the Nan-D group was mainly enriched with *g__Paraprevotella* and *g__Anaerostipes*. Collectively, from the perspectives of differential taxa and group-specific biomarkers, these findings reveal that the various interventions differentially modulated the gut microbiota structural alterations induced by the HFD.

##### Correlation Analysis Between Gut Microbiota and Lipid Metabolism Indicators

Spearman correlation analysis was performed to assess associations between significantly enriched gut bacteria and lipid metabolism parameters (Figure 9c). *Clostridioides difficile*, *Enterococcus clostridioformis*, and *Ruminococcus gnavus* demonstrated significant positive correlations with serum TC, TG, HbA1c, and TBA levels (*p* < 0.05), but significant negative correlations with short-chain fatty acid (SCFA) related indicators. Conversely, *Mycoplasma*_sp. CAG 877 exhibited significant negative correlations with TC, TG, LDL-C, HbA1c, and TBA, alongside significant positive correlations with SCFA-related indicators. *Candidatus* Scatovivens faecipullorum was also significantly negatively correlated with TC, TG, HbA1c, and serum TBA, while showing significant positive correlations with acetate and total SCFA concentrations (*p* < 0.05). Furthermore, *Lactobacillus helveticus*, *Limosilactobacillus reuteri*, *Limosilactobacillus vaginalis*, *Candidatus Coprovivens excrementavium*, and *Clostridium*_sp. CAG 452 all displayed significant negative correlations with serum TBA (*p* < 0.05).

##### Differences in Gut Microbiota Functional Composition

Statistically significant intergroup differences in KEGG pathway abundance were identified using the Kruskal–Wallis H test (*p* < 0.05). The results indicated that under carbohydrate metabolism, pathways including glycolysis/gluconeogenesis (*p* = 0.01746) and galactose metabolism (*p* = 0.04997) showed significant distribution differences across groups. Within amino acid metabolism, histidine metabolism (*p* = 0.03434) and valine, leucine and isoleucine biosynthesis (*p* = 0.01346) also exhibited significant intergroup variations. Among pathways directly related to hyperlipidemia, the fatty acid degradation pathway (*p* = 0.03268) under lipid metabolism displayed significant intergroup differences in abundance. In addition, within the endocrine system and endocrine/metabolic disease categories, the PPAR signaling pathway (*p* = 0.01185) and the lipid and atherosclerosis pathway (*p* = 0.00751) showed particularly pronounced intergroup differences in abundance. These differentially regulated pathways are implicated in host energy metabolism, lipid degradation, and the regulation of atherosclerosis-related pathological processes. Collectively, the findings suggest that the probiotic complex may modulate lipid metabolism disorders and related pathological states in hyperlipidemic rats by reshaping the functional pathway profile of the gut microbiota.

#### 2.4.2. Effects of Composite Probiotics on SCFA Levels

This study further examined the effects of the Equine Colostrum-derived composite probiotic on fecal SCFA levels in hyperlipidemic rats. SCFAs, primary metabolites of gut microbiota from dietary fiber fermentation, predominantly accumulate in the colon where they modulate cholesterol metabolism, free fatty acid homeostasis, and glucose regulation to attenuate adiposity [15]. As shown in Table 2, fecal acetate, propionate, butyrate, and total SCFA concentrations were significantly lower in Group M than in Group N (*p* < 0.01), suggesting compromised microbial metabolic function. Following intervention, both the PC and Nan-G groups exhibited significantly elevated levels of acetate, butyrate, and total SCFAs compared to Group M (*p* < 0.01).

#### 2.4.3. Effects of Composite Probiotics on Bile Acid Levels

The effects of the composite probiotic on bile acid metabolism in hyperlipidemic rats are presented in Table 2. Compared to Group N, Group M exhibited significantly elevated total TBA levels in serum and feces (*p* < 0.01), indicating HFD-induced disruption of bile acid homeostasis. Post-intervention, serum TBA levels decreased significantly in the PC and Nan-G groups relative to Group M (*p* < 0.01). Concurrently, fecal TBA levels were significantly reduced across all intervention groups (*p* < 0.01). These findings suggest that the probiotic may facilitate the restoration of bile acid homeostasis by modulating enterohepatic circulation and influencing hepatic synthesis, thereby contributing to systemic lipid regulation.

#### 2.4.4. Correlation Analysis of Lipid-Related Parameters, Short-Chain Fatty Acids and Bile Acids

**Figure 10:**
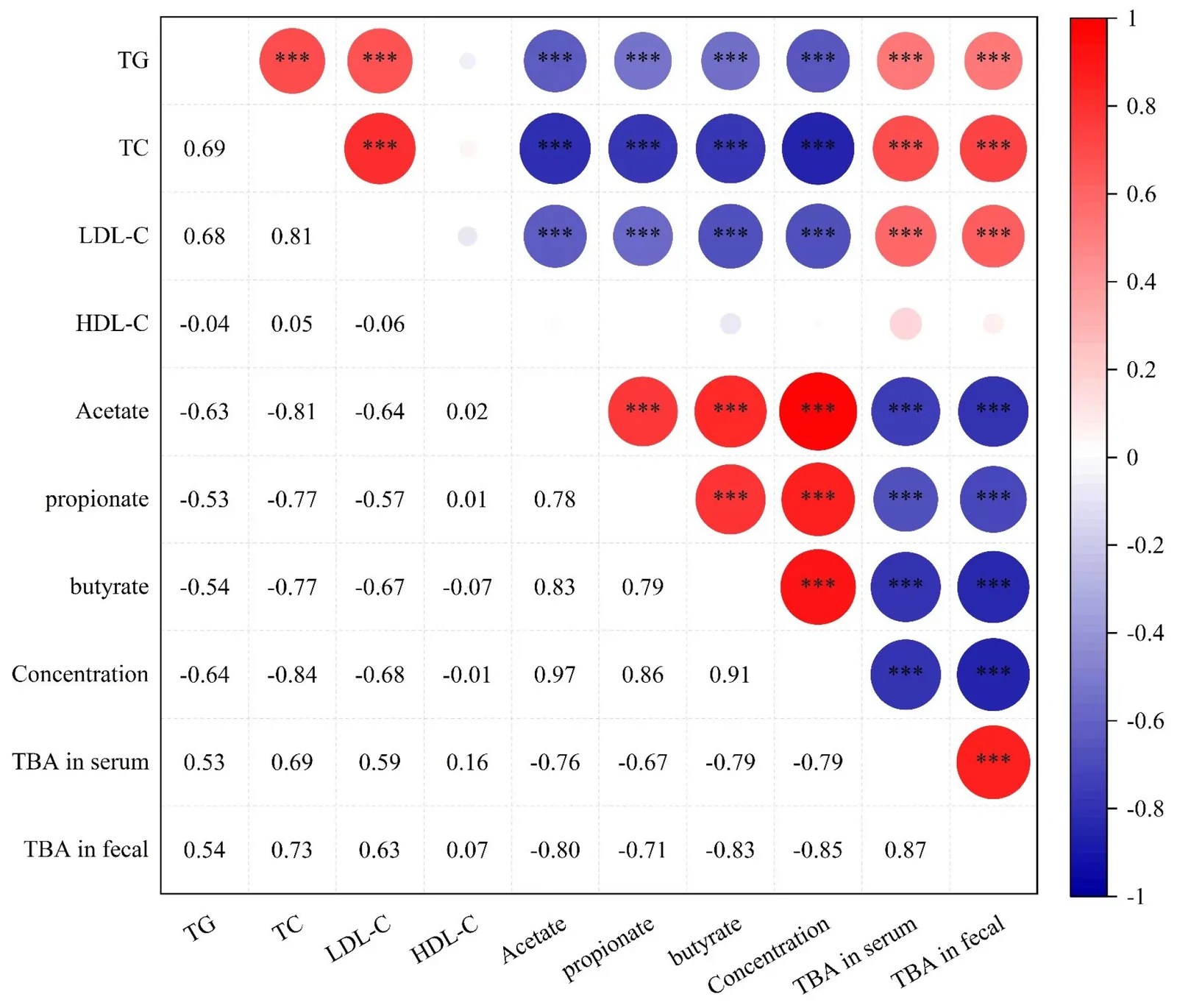
Correlation heat-map among serum lipid indicators, short-chain fatty acids and bile-acid parameters. Inside each circle, *** *p* < 0.001. Red circles indicate positive correlations, whereas blue circles represent negative correlations. The color intensity reflects the magnitude of the correlation coefficient. TG: triglyceride; TC: total cholesterol; LDL-C: low-density lipoprotein-cholesterol; HDL-C: high-density lipoprotein-cholesterol; TBA in serum: serum total bile acids; TBA in feces: fecal total bile acids.

To further explore the intrinsic correlations among serum lipid profiles, SCFAs, and bile acids, Spearman correlation analysis was performed in the present study. As shown in Figure 10, serum TC, TG and LDL-C exhibited extremely significant positive correlations with one another (*p* < 0.001), indicating consistent changing trends among these lipid indicators in rats. No significant correlation was observed between HDL-C and the other serum lipid parameters. Acetate, propionate, butyrate, and total SCFA concentration were significantly and negatively correlated with TG, TC, and LDL-C (*p* < 0.001), demonstrating that elevated SCFA levels were closely associated with reduced serum lipid levels. In addition, acetate, propionate, butyrate, and total SCFA concentration were mutually positively correlated (*p* < 0.001), suggesting synchronous variation patterns of the three SCFA subtypes.

SCFA levels were significantly negatively correlated with both serum and fecal TBA (*p* < 0.001), while serum TBA levels were positively correlated with fecal total bile acid levels (*p* < 0.001). Moreover, TG, TC, and LDL-C presented moderate positive correlations with serum and fecal total bile acids. Collectively, increased SCFA contents were closely linked to decreased lipid and bile acid levels. The SCFA–bile acid axis may serve as a critical regulatory pathway participating in the modulation of lipid metabolism in hyperlipidemic rats.

## 3. Discussion

Residents in the pastoral regions of Xinjiang have a long-standing tradition of consuming fermented dairy products, which harbor diverse microorganisms and have been reported to offer health benefits, including blood lipid regulation. These products constitute a unique repository of microbial resources, their value derived from the variety of locally adapted strains. The probiotic properties of these beneficial bacteria are often highly strain-specific, and their functions are influenced by multiple factors, such as milk source type, chemical composition, geographic origin, and environmental conditions [20]. As one of the earliest regions for horse domestication, Xinjiang preserves a rich diversity of probiotic resources in Equine Colostrum products. However, with advancing industrialization, some traditional strains face the risk of endangerment or even extinction, making in-depth research on them urgently needed [21].

**Figure 11:**
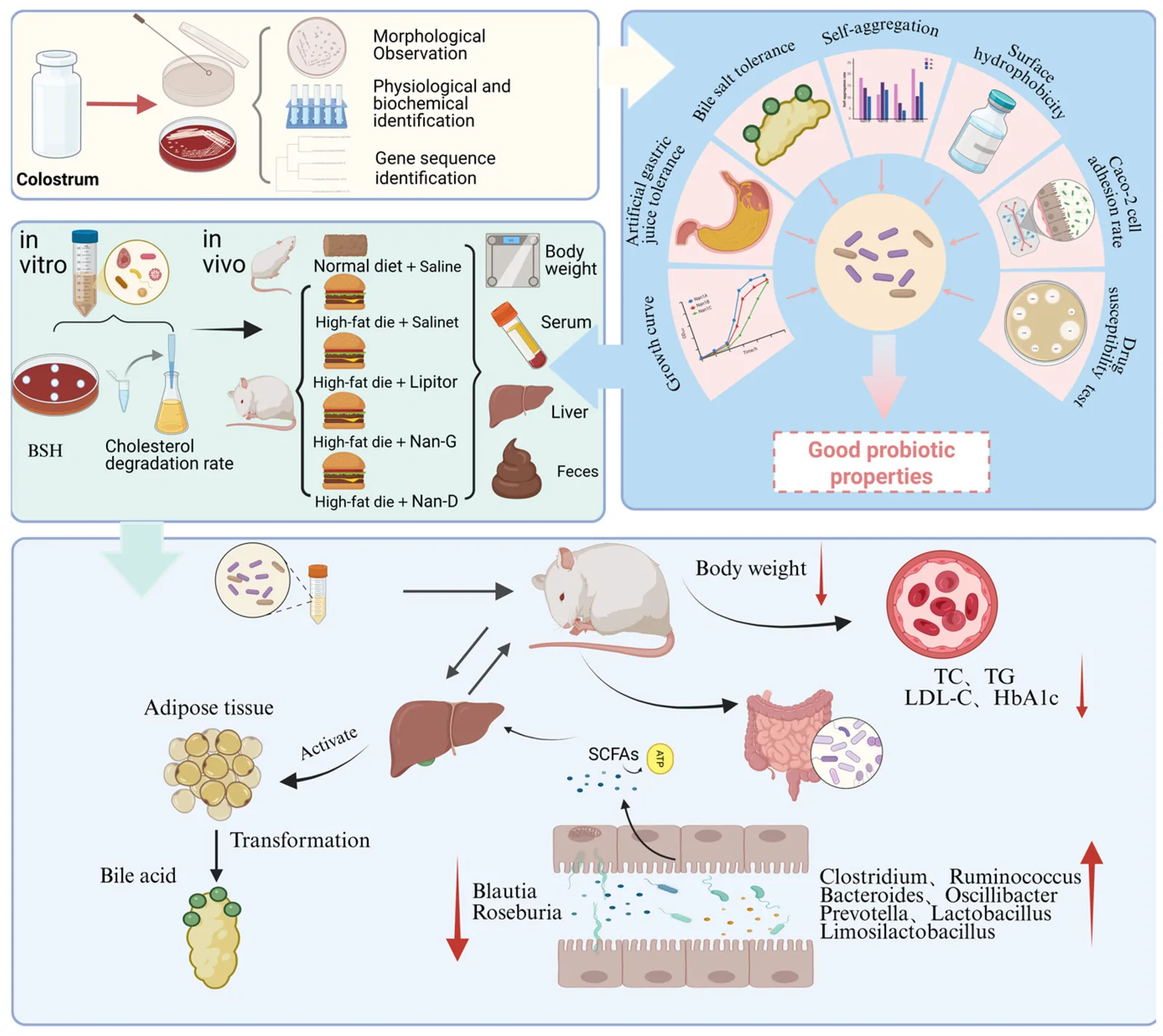
Isolation and Identification of Multi-Strain Probiotics from Equine Colostrum and Their Impact on Serum Lipid Profiles. An upward-pointing arrow in the figure indicates that the index increases after intervention, while a downward-pointing arrow indicates that the index decreases after intervention.

As shown in Figure 11, A total of 10 LAB strains and 6 yeast strains were isolated from equine colostrum samples collected in the pastoral regions of Xinjiang. Based on their probiotic properties and in vitro cholesterol-lowering capacities, five LAB and two yeast strains were selected for subsequent investigation. Comprehensive evaluations, encompassing growth kinetics, gastrointestinal tolerance, mucosal adhesiveness, safety, and cholesterol-reduction efficacy, collectively substantiated their probiotic potential. Specifically, all seven strains exhibited markedly high survival rates in simulated gastric juice (pH 2.0) and intestinal fluid containing 3.0 g/L bile salts, indicating a robust capacity to traverse the gastrointestinal tract and establish intestinal colonization. This observation is consistent with prior reports demonstrating that *Lactiplantibacillus plantarum* isolated from traditional fermented foods maintains high viability under simulated gastrointestinal conditions [22]. Auto-aggregation assays revealed that the self-aggregating ability of all tested strains increased progressively over time, accompanied by gradual clarification of the supernatant, which suggested a pronounced intercellular aggregation tendency. In terms of cell-surface hydrophobicity, the strains displayed moderate affinity for xylene, with values ranging from 12.56% to 55.36%, a range that aligns with the moderate hydrophobicity spectrum (25.56–69.93%) previously documented for LAB [23]. Furthermore, all strains demonstrated adhesion to Caco-2 cells, further supporting their potential for effective intestinal colonization and competitive exclusion of enteropathogens. Safety constitutes a fundamental prerequisite for the application of probiotics in humans. Antibiotic susceptibility testing indicated intrinsic resistance to vancomycin and ciprofloxacin, corroborating earlier findings [15,23]. Notably, the majority of strains remained susceptible to multiple clinically prevalent antibiotics, thereby reinforcing their favorable safety profile.

In terms of cholesterol-removal capacity, all five lactic-acid-bacteria strains exhibited BSH activity and were capable of reducing cholesterol concentrations in culture medium. Nevertheless, the strain with the highest BSH activity did not show the strongest cholesterol-removal efficiency, indicating that the cholesterol lowering mechanism is not solely dependent on the BSH-mediated pathway, and may additionally involve direct bacterial assimilation, co-precipitation or other metabolic routes [17]. We speculate that in vitro cholesterol elimination encompasses multiple mechanisms, including BSH-driven bile acid deconjugation and co-precipitation, cell-wall adsorption by bacterial cells, as well as cholesterol assimilation into cell membranes. Even for strains with the strongest BSH activity, insufficient cell-surface hydrophobicity or weak adsorption capacity may prevent them from achieving optimal overall cholesterol-removal performance [18]. Conversely, strains with moderate BSH activity but superior membrane fluidity or assimilation efficiency could compensate for limited BSH activity via adsorption and assimilation pathways, thus attaining a higher total cholesterol-removal rate. A multi-indicator comprehensive evaluation system will be established in our subsequent research [24]. Although the composite probiotic preparation did not yield the highest cholesterol-removal rate in the in vitro assay, all strains included in this formulation were isolated from the identical mare-colostrum sample, which inherently provides a biological foundation for their co-existence and mutual interaction. More importantly, consistent with our group’s previous research on camel-milk-derived probiotics and published literature, multi-strain probiotic consortia usually exert better physiological-regulatory effects than single strains. The combined strains function analogously to a “joint-force army” to perform their modulatory roles. A prominent mutual-beneficial relationship exists between yeasts and lactic-acid bacteria within this composite system: during fermentation, yeasts supply multiple nutritional factors such as amino acids, vitamins and pyruvate for lactic-acid bacteria, and secrete proteases and lipases to decompose substrates and generate growth-promoting substances. In return, metabolic end-products from lactic-acid bacteria serve as energy sources for yeasts, forming metabolically complementary symbiosis. This symbiotic interaction endows the multi-strain consortium with the advantages of “co-operative operation” [9,19]. Single strains frequently suffer from weak intestinal colonization capacity, susceptibility to interference from resident gut microbiota and limited functional targets under in vivo conditions. Their outstanding in vitro performance is often difficult to reproduce inside the host. By contrast, multi-strain combinations can overcome these drawbacks through synergistic interactions and achieve improved lipid-lowering efficacy [9]. Taken together, these findings demonstrate that the seven isolated strains possess favorable gastrointestinal tolerance, strong epithelial-cell adhesion potential and reliable safety profiles. Hence, they are promising probiotic candidates for the development of functional foods or related preparations. Further in vivo animal experiments are required to validate their lipid-lowering efficacy and uncover their underlying functional mechanisms.

The selected strains were formulated into a composite probiotic preparation and subsequently administered to a hyperlipidemic rat model over an 8-week intervention period, during which systematic evaluations were conducted on serum lipid profiles, glycemic indices, hepatic histopathology, and gut microbiota composition. The results demonstrated that probiotic intervention significantly attenuated high-fat diet (HFD)-induced weight gain and reduced serum levels of total cholesterol (TC), triglycerides (TG), low-density lipoprotein cholesterol (LDL-C), and glycated hemoglobin (HbA1c), suggesting a dual modulatory effect on both lipid and glucose metabolic homeostasis, a finding that aligns with previous reports [25]. Hepatic histological examination further revealed that probiotic treatment markedly alleviated intrahepatic lipid deposition and pathological damage induced by HFD, potentially through the modulation of hepatic lipid metabolic pathways.

The role of gut microbiota in human health and disease has been extensively documented [26]. Probiotic supplementation can modulate gut microbial composition, thereby influencing host dietary lipid metabolism. Our results showed that the HFD reduced microbial diversity and elevated the Firmicutes/Bacteroidetes (F/B) ratio—a marker commonly associated with obesity and metabolic dysregulation [27]—whereas probiotic intervention effectively reversed these alterations. In the intervention group, beneficial genera such as *Ruminococcus*, *Bacteroides*, *Oscillospira*, as well as multiple *Lactiplantibacillus* and *Limosilactobacillus* species, were enriched. Among these, *Bacteroides* and *Ruminococcus* are known short-chain fatty acid (SCFA) producers [28]. Notably, *Oscillospira* abundance was increased in the positive-control (atorvastatin) group but did not change significantly in the probiotic group, suggesting differential modulatory effects on this genus between the two interventions [29,30]. LEfSe analysis further revealed that the HFD group was enriched in *Lachnospiraceae* and *Blautia*—the latter belonging to the *Lachnospiraceae* family and positively associated with metabolic disorders. *Blautia* has been reported to participate in acetate and butyrate production [31]; however, previous studies have also shown increased abundance of this genus in obese individuals and in HFD-induced type 2 diabetic rats, implying a potential link with metabolic perturbation [32]. In contrast, the probiotic group was specifically enriched in *Limosilactobacillus* and *Lactiplantibacillus*, with the latter’s enrichment being consistent with its known role in enhancing bile salt hydrolase activity and improving lipid metabolism [33].

Metagenomic analysis revealed that probiotic administration significantly altered the abundance of pathways involved in carbohydrate, amino acid, and lipid metabolism. Metabolite profiling confirmed that the HFD reduced fecal levels of acetate, propionate, and butyrate, whereas probiotic intervention significantly elevated acetate and butyrate concentrations, a change that was consistent with the shifts observed in SCFA-producing bacteria. Our findings are in agreement with those of Li et al. [34], who demonstrated that combined probiotics can regulate host lipid metabolism by remodeling gut microbial structure and enhancing acetate and butyrate levels. Furthermore, the HFD-induced increase in serum and fecal total bile acids (TBA) indicated a disturbance in enterohepatic circulation, while probiotic treatment effectively lowered TBA levels, thereby reflecting the bidirectional regulatory interplay between the gut microbiota and bile acid metabolism [35].

Spearman’s correlation analysis was performed in this study to investigate the relationships among serum lipidrelated parameters, SCFAs and TBA. The results revealed that TG, TC, LDL-C were significantly negatively correlated with SCFA concentrations. Strong negative correlations were observed between SCFA levels and TBA concentrations in both serum and feces, whereas lipid levels were positively correlated with bile-acid contents. These correlation findings further substantiated the existence of a “short-chain fatty acid–bile acid–lipid metabolism” regulatory axis, providing supplementary evidence for the cholesterol-lowering pathways mediated by the composite probiotic preparation.

Taken together, the composite probiotic preparation synergistically improved lipid and glucose metabolic homeostasis and alleviated intrahepatic lipid accumulation in hyperlipidemic rats, primarily via remodeling gut microbial architecture and modulating short-chain fatty acid and bile acid metabolism. These results provide experimental evidence supporting its potential for development as a functional food or pharmaceutical agent. Nevertheless, the precise synergistic mechanisms underlying these beneficial effects remain to be further elucidated.

Nevertheless, several notable limitations of the present study should be acknowledged. First, only the BSH-mediated pathway was verified among the in vitro cholesterol lowering mechanisms. Other potential mechanisms including cholesterol assimilation, co-precipitation with bile salts and bacterial cell adsorption have not been experimentally validated in the current work, which will be systematically investigated in follow up research. Second, the mRNA or protein expression levels of key genes involved in lipid metabolism were not directly measured, and thus molecular evidence supporting the cholesterol lowering mechanism remains insufficient. Reasonable inferences were drawn on the basis of published literature and correlation results, and subsequent direct validation experiments using qRT-PCR or Western blot will be carried out. Third, KEGG functional enrichment analysis was predicted from metagenomic data without functional verification via enzyme activity assays, metabolomics or transcriptomics. Targeted metabolomics will be adopted in future studies to cross validate these predicted pathways. Fourth, only the overall efficacy of the composite probiotic preparation was evaluated in the in vivo animal trial. Separate in vivo tests for individual high-activity strains screened in vitro were not performed. Further systematic comparison of single strains and different mixed combinations will be conducted to clarify their independent contributions and synergistic effects.

In future work, targeted pathway blocking experiments combined with multi omics analyses will be implemented to quantitatively determine the relative contribution of each lipid lowering pathway, clarify the synergistic regulatory mechanisms among these pathways, and comprehensively unravel the molecular cholesterol lowering mechanism of this multi-strain probiotic consortium.

## 4. Materials and Methods

### 4.1. Reagents and Instruments

Reagents and kits for microbiological characterization, including a Gram staining kit, 3% (*w*/*v*) hydrogen peroxide (catalase reagent), oxidase test strips, gelatin biochemical tubes, and indole reagent kit, were purchased from Haibo Biotechnology Co., Ltd. (Qingdao, China). Biochemicals such as sodium taurocholate, sodium thioglycolate, anhydrous CaCl_2_, bovine bile salts, cholesterol, and pepsin (for preparing simulated gastric fluid) were obtained from Shanghai Yuanye Biotechnology Co., Ltd. (Shanghai, China). Antibiotic susceptibility test disks were sourced from Wenzhou Kangtai Biological Technology Co., Ltd. (Wenzhou, China). Culture media, including de Man, Rogosa and Sharpe (MRS) broth and agar, Malt Extract Broth and Malt Extract Agar, were purchased from Beijing Solarbio Technology Co., Ltd. (Beijing, China). Trypticase–Phytone–Yeast (TPY) agar was obtained from Haibo Biotechnology Co., Ltd. Assay kits for quantifying serum triglycerides (TG), total cholesterol (TC), high-density lipoprotein cholesterol (HDL-C), low-density lipoprotein cholesterol (LDL-C), and total bile acids (TBA) were purchased from Shenzhen Mindray Bio-Medical Electronics Co., Ltd. (Shenzhen, China) and Shanghai Youxuan Biotechnology Co., Ltd. (Shanghai, China), respectively. Additional chemicals and cell culture reagents included xylene, ethyl acetate, DMEM medium, Caco-2 cell-specific medium, atorvastatin calcium (Lipitor^®^, Pfizer, Dalian, China), Acetic Acid, Propionic Acid, Butyric Acid, 2-Ethylbutyric Acid.

All instruments used in this study are listed as follows. For sample preparation and processing, we employed an electronic analytical balance (ME204, Mettler Toledo, Shanghai, China), a constant-temperature water bath (DK-8D, Jinghong, Shanghai, China), and a steam sterilizer (SX-500, Tomy Digital Biology, Tokyo, Japan). Microbial culture and observation were conducted using an anaerobic incubator (YQX-III, Xinmuo Instrument, Shanghai, China) and a biological microscope (XSP-BM-2CA, Shangguang, Ningbo, China). For sample separation and analysis, a high-speed refrigerated centrifuge (CHT210R, Xiangyi Centrifuge, Changsha, China), a microplate reader (Multiskan GO, Thermo Fisher Scientific, Waltham, MA, USA), a fully automatic biochemical analyzer (BS-120, Mindray, Shenzhen, China), and a gas chromatograph (GC-2010 Plus, Shimadzu, Kyoto, Japan) were utilized.

### 4.2. Sample Collection and Research Subjects

#### 4.2.1. Sample Source

Sample size: Three independent mares were selected, and sample collection was performed in two batches. The collected mare colostrum was aliquoted into 50 mL sterile centrifuge tubes, sealed and labeled, transported at low temperature in an ice box, and delivered to the laboratory within 4 h. One portion of the samples was immediately used for strain isolation and culture; the residual samples were frozen and stored at −80 °C in a refrigerator for subsequent use.

Sampling site: Samples were collected from different pastoral households in the Nanshan Pastoral Area, Urumqi. Before sampling, the teat surface of each mare was thoroughly disinfected to avoid exogenous contamination.

Biological replicates: Colostrum samples collected from different individual mares were defined as biological replicates. Multiple parallel isolation experiments conducted on colostrum from the same mare were only regarded as technical controls and not counted as biological replicates.

Selection criteria: Healthy mares within 24 h postpartum with no mastitis or other systemic diseases were enrolled. The mares were 5–6 years old, with a body weight of 250–300 kg.

#### 4.2.2. Laboratory Animals

Forty male Sprague-Dawley (SD) rats, aged 6 weeks and of specific pathogen-free (SPF) grade, were obtained from the Animal Experiment Center of Xinjiang Medical University. The body weight of the rats ranged from 180 to 200 g. The experimental protocol was approved by the Institutional Animal Care and Use Committee (IACUC) of Xinjiang Medical University (Approval No. IACUC-20250720-01). All procedures were conducted in accordance with the National Guidelines for Animal Welfare. The animals were supplied under the following licenses: Production License SCXK(Xin)2023-0001 and Use License SYXK(Xin)2023-0004. Animals were housed under standard conditions with a 12 h light/dark cycle, ambient temperature maintained at 20–26 °C, and relative humidity at 40–70%. Food and water were available ad libitum. Bedding was changed twice per week.

### 4.3. Research Methods

#### 4.3.1. Isolation, Purification, and Identification of Strains

##### Isolation and Purification of Strains

The collected colostrum samples were homogenized and serially diluted with sterile physiological saline. Aliquots (100 µL) from appropriate dilutions were spread-plated onto MRS and TPY agar plates. Plates were then incubated anaerobically at 37 °C for 24–48 h. Following incubation, individual colonies were selected from plates containing mixed growth and purified by repeated streaking on fresh agar. Purified colonies were subjected to Gram staining; those that were Gram-positive and catalase-negative were preliminarily classified as lactic acid bacteria [36]. In parallel, the homogenized colostrum samples were also plated on Shah’s agar medium and incubated at 25 °C for 48–72 h. Distinct single colonies were examined microscopically, purified via the streak plate method, and subsequently identified based on their physiological and biochemical characteristics.

##### Identification of Strains

Colony morphology was observed and characterized based on size, texture, elevation, margin, transparency, surface smoothness, luster, and color. Physiological and biochemical identification of lactic acid bacteria was subsequently performed, including Gram staining, catalase activity, oxidase activity, gelatin liquefaction, indole production, growth at 45 °C, tolerance to 6.5% NaCl, and fermentation tests with various sugars and carbon sources. For yeast strains, isolates were inoculated into Malt Extract broth, Malt Extract Agar, and Cornmeal Agar (Dalmau plate culture) to assess growth characteristics; biochemical identification was performed in parallel with carbohydrate fermentation tests. Finally, representative strains were submitted to the Institute of Microbiology, Chinese Academy of Sciences (IMCAS) for 16S rRNA (bacterial) or 26S rRNA (yeast) gene sequencing for definitive taxonomic identification.

#### 4.3.2. Preparation of Culture Media and Bacterial Suspensions

##### MRS-CHOL Medium

A cholesterol stock solution (10 g/L) was prepared by dissolving 1.0 g of cholesterol in anhydrous ethanol and bringing the final volume to 100 mL in a volumetric flask. This solution was then subjected to filter-sterilization using a 0.22 µm microporous membrane under aseptic conditions. Separately, MRS broth was supplemented with 0.3% (*w*/*v*) bile salts and sterilized by autoclaving at 121 °C for 20 min. Finally, the filter-sterilized cholesterol stock solution was aseptically added to the autoclaved broth to achieve a final cholesterol concentration of 1.0 g/L in the medium.

##### Preparation of Bacterial Suspension

Five strains of lactic acid bacteria and two strains of yeast were cultured separately in MRS broth and Malt Extract broth, respectively, at 37 °C for 48 h. Following centrifugation, the cells were harvested, washed twice with sterile physiological saline, and resuspended in an appropriate buffer. The concentrations of the five lactic acid bacterial strains were adjusted to 1 × 10^10^ CFU/mL and 1 × 10^8^ CFU/mL, while those of the two yeast strains were adjusted to 1 × 10^8^ CFU/mL and 1 × 10^6^ CFU/mL using the plate colony counting method. Subsequently, equal volumes of the adjusted suspensions were combined to constitute two composite microbial consortia: a high-dose consortium containing the five lactic acid bacteria strains (1 × 10^10^ CFU/mL) and the two yeast strains (1 × 10^8^ CFU/mL), and a low-dose consortium containing the five lactic acid bacteria strains (1 × 10^8^ CFU/mL) and the two yeast strains (1 × 10^6^ CFU/mL). All composite consortia were freshly prepared prior to experimental use.

#### 4.3.3. Probiotic Characteristic Evaluation

##### Growth Curve Measurement

The activated bacterial strains were inoculated at 3% (*v*/*v*) into MRS broth and Malt Extract broth and then incubated for 72 h. The optical density at 600 nm (OD_600_) was measured in triplicate. Growth curves for each strain were subsequently plotted using incubation time (h) as the independent variable (*x*-axis) and the mean OD_600_ as the dependent variable (*y*-axis).

##### Assessment of Bile Salt Tolerance

Bovine bile salts were added to MRS broth and Malt Extract broth to a final concentration of 3.0 g/L, followed by sterilization and cooling to 30–40 °C. The activated bacterial strains were inoculated at 3% (*v*/*v*) into the bile salt-supplemented media; bile salt-free media served as controls. Following a 3 h incubation under the respective conditions, the OD_600_ was measured with three independent biological replicates per group. The bile salt tolerance rate was then calculated using Equation (1).

(1) $$\mathrm{Tolerance}\;\mathrm{rate}/\%=[{\mathrm{OD}}_{600}\;(\mathrm{bile}\;\mathrm{salt}\text{-}\mathrm{treated}\;\mathrm{culture})/{\mathrm{OD}}_{600}\;(\mathrm{untreated}\;\mathrm{control})]\;\times \;100$$

##### Assessment of Tolerance to Simulated Gastric Fluid

The activated bacterial strains were inoculated into their respective media and then incubated anaerobically for 24 h at their designated growth temperatures. After cultivation, bacterial cells were harvested by centrifugation at 6123× *g* for 10 min at 4 °C. The pellet was washed twice with phosphate-buffered saline (PBS, pH 7.2) and finally resuspended in artificial gastric juice (pH 2.0) containing 3.2 g/L pepsin for subsequent tolerance assays. The OD_600_ of the cultures was measured at 0 and 3 h. All experiments were conducted in triplicate, and the corresponding survival rates were calculated according to Equation (2).

(2) $$\mathrm{Tolerance}\;\mathrm{rate}/\%=[{\mathrm{OD}}_{600}\;(3\;h)/{\mathrm{OD}}_{600}\;(0\;h)]\;\times \;100$$

##### Auto-Aggregation Assay

Following the method described by Xiao Chunjin et al. [37], the activated bacterial suspension was centrifuged at 4 °C and 3444× *g* for 10 min to collect the bacterial cells. The cells were washed with PBS and resuspended to a concentration of 1 × 10^8^ CFU/mL, and the optical density at OD_600_ of the suspension (*A*_0_) was measured. A 4 mL aliquot of the bacterial suspension was incubated at 37 °C. The supernatants were collected at 1, 2, 4, 8, and 24 h, and the OD_600_ value (*A*_t_) was determined for each time point. All experiments were performed in triplicate. The self-aggregation rate was calculated using Equation (3).

(3) $$\mathrm{Self}\text{-}\mathrm{aggregation}\;\mathrm{rate}/\%=(1-A_{t}/A_{0})\;\times \;100$$

In the equation, *A*_0_ represents the OD_600_ at 0 h, and *A*_t_ corresponds to the OD_600_ measured at 1, 2, 4, 8,and 24 h, respectively.

##### Determination of Surface Hydrophobicity

Bacterial suspensions were prepared as described in Section Auto-aggregation Assay. For each solvent (xylene or ethyl acetate), 3 mL of a bacterial suspension was mixed with 1 mL of the solvent, vortexed thoroughly, and the initial optical density at OD_600_, recorded as *C*_0_) was measured immediately. The mixtures were then incubated at 37 °C for 2 h to allow phase separation. After incubation, the OD_600_ of the aqueous phase (*C*_i_) was measured. Three independent replicates were performed for each solvent treatment. The surface hydrophobicity was calculated using Equation (4).

(4) $$\mathrm{Surface}\;\mathrm{hydrophobicity}/\%=(1-C_{i}/C_{0})\times \;100$$

In the equation, *C*_0_ represents the OD_600_ at 0 h, and *C*_i_ represents the OD_600_ at 2 h.

##### Caco-2 Cell Adhesion Rate Assay

Bacterial adhesion to Caco-2 cell was evaluated according to the method described by Chen et al. [38]. Briefly, Caco-2 cells were seeded in 12-well plates at a density of 1 × 10^5^ cells/mL and cultured to full confluency. The cell monolayers were washed twice with PBS. Subsequently, each well was inoculated with 0.5 mL of a bacterial suspension (1 × 10^8^ CFU/mL), prepared in Dulbecco’s Modified Eagle Medium. Following incubation at 37 °C for 2 h, non-adherent bacteria were removed by washing three times with PBS. Adherent bacteria along with the Caco-2 cells were then detached by treatment with trypsin, and the digestion was stopped by adding complete culture medium. The collected suspension was serially diluted, plated on appropriate agar media, and incubated for viable bacterial enumeration. The adhesion rate was calculated using Equation (5). All assays were performed with three independent replicates for each bacterial strain.

(5) Adhesion rate/%= Number of adherent viable bacteria/Initial number of viable bacteria added×100

##### Safety Evaluation of Strains

Antimicrobial susceptibility of the strains was determined using the Kirby–Bauer disk diffusion method [39], in accordance with the Clinical and Laboratory Standards Institute (CLSI) M100 guidelines (Supplementary Table S6). For lactic acid bacteria, 100 µL aliquots of suspension (1 × 10^8^ CFU/mL) were aseptically spread onto MRS agar plates. Four distinct antibiotic disks were then applied to each plate. Plates were incubated anaerobically at 37 °C for 24–48 h. For yeast, 100 µL aliquots of suspension (1 × 10^5^ CFU/mL) were similarly processed by spreading onto Malt Extract Agar plates, followed by the application of four distinct antifungal disks. Yeast plates were incubated at 27 °C for 48–72 h under the atmospheric conditions specified by the CLSI guideline for this medium. Following incubation, the diameters of the inhibition zones were measured using a digital caliper with a precision of ±0.1 mm. All assays were performed in three independent biological replicates.

#### 4.3.4. In Vitro Cholesterol-Lowering Effect

##### Qualitative Assay of BSH Activity

Sodium taurocholate (3.0 g/L), sodium mercaptoacetate (2.0 g/L), and anhydrous CaCl_2_ (0.37 g/L) were incorporated into MRS agar. After sterilization by autoclaving, the medium was aseptically poured into Petri dishes. Sterile filter paper disks were immersed in bacterial suspensions adjusted to 1 × 10^8^–1 × 10^9^ CFU/mL. The disks were then placed on the agar surface, and the plates were incubated anaerobically at 37 °C for 72 h. A positive BSH reaction was indicated by the formation of opaque, granular colonies or a precipitation halo with a radius of ≥1 mm surrounding the disk.

##### The Analysis of in Vitro Cholesterol Degradation Rate

The in vitro cholesterol degradation capacity of the strains was evaluated according to Yang et al. [40]. Lactic acid bacterial suspensions, yeast suspensions, and low- and high-dose composite probiotic suspensions were inoculated at 3% (*v*/*v*) into their respective broth media supplemented with cholesterol (1.0 g/L). Cholesterol-free media were used as blank controls. All cultures were incubated at 37 °C for 48 h. After incubation, 250 µL of the enzyme working solution (cholesterol oxidase/peroxidase) was mixed separately with 2.5 µL aliquots of the following: sample supernatant, blank control solution, 6.55 mmol/L cholesterol standard solution, and distilled water. The resulting mixtures were incubated at 37 °C for 10 min, and the absorbance at 500 nm (OD_500_) was measured. Each sample was assayed in triplicate. The cholesterol degradation rate was calculated using Equations (6) and (7).

(6) $$\mathrm{Cholesterol}/(\mathrm{mmol}\cdot L^{-1})=\;6.55\;\times {\;(S}_{1}-S_{2})\;/\;{(S}_{\mathrm{standard}}-S_{0})$$

In the equation, *S*_1_ is the OD_500_ value of the test sample; S_2_ is the OD_500_ value of the blank control; *S*_standard_ is the OD_500_ value of the standard; *S*_0_ is the OD_500_ value of distilled water.

(7) $$\mathrm{Cholesterol}\;\mathrm{degradation}\;\mathrm{rate}/\%=\;(X_{0}-X_{1})/\;X_{0}\times \;100$$

In the equation, *X*_0_ is the cholesterol content (mmol/L) in the culture medium before inoculating the test bacteria; *X*_1_ is the cholesterol content (mmol/L) in the culture medium after 48 h of bacterial inoculation.

#### 4.3.5. In Vivo Cholesterol-Lowering Effect

##### Animals and Treatments

Forty specific pathogen-free (SPF) male Sprague-Dawley (SD) rats were acclimatized for one week and then randomly divided into a normal control group (N; n = 8) and a hyperlipidemia model group (n = 32). The normal group was fed a standard basal diet for growth and reproduction, while the model group received a 60% high-fat diet (HFD) (D12492). The model was considered successfully established when the serum levels of triglycerides (TG), total cholesterol (TC), and low-density lipoprotein cholesterol (LDL-C) in the model group were significantly higher than those in the normal group (*p* < 0.05). Subsequently, rats with successful hyperlipidemia modeling were randomly assigned into the following groups (n = 8 per group): model group (M), positive-control group (PC, atorvastatin calcium, 8 mg/kg), high-dose composite probiotic group (Nan-G), and low-dose composite probiotic group (Nan-D). The intragastric gavage volume was set at 10 mL/kg·d. Animal body weights were recorded every Tuesday morning, and the gavage dosage was adjusted weekly on this basis. For the treatment groups, Nan-G was administered a daily live bacterial preparation containing lactic acid bacteria at 1 × 10^10^ CFU/mL and yeast at 1 × 10^8^ CFU/mL; Nan D received lactic acid bacteria at 1 × 10^8^ CFU/mL and yeast at 1 × 10^6^ CFU/mL. The normal control group (N) and model group (M) were intragastrically administered an equal volume of normal saline every day. The positive control group (PC) received atorvastatin calcium at a daily dose of 8 mg/kg. All intragastric interventions were carried out for 8 consecutive weeks.

##### Sample Collection and Processing

After 8 weeks of gavage feeding, the rats were fasted for 12 h. Following body weight measurement, they were anesthetized by intraperitoneal injection of an anesthetic, and blood was collected from the abdominal aorta. The blood samples were allowed to clot at room temperature for 2 h, then centrifuged at 861× *g* for 15 min. The separated serum was aliquoted into sterile centrifuge tubes and stored at −80 °C pending analysis. Immediately after blood collection, the entire intestine was excised. Under sterile conditions, the luminal contents were gently flushed and transferred into cryotubes for rapid freezing in liquid nitrogen, followed by long-term storage at −80 °C. For liver tissue, one portion was fixed in paraformaldehyde for histological sectioning, while the remainder was snap-frozen and stored at −80 °C for subsequent analysis.

##### Biomarker Quantification

Serum levels of TC, TG, LDL-C, and HDL-C were measured using a fully automated biochemical analyzer. Glycated hemoglobin and total bile acids were determined using enzyme-linked immunosorbent assay (ELISA) kits according to the manufacturer’s protocols.

##### Liver Pathology

Upon completion of the experimental protocol, rat livers were excised to assess histopathological alterations across all experimental groups. For histological analysis, tissue samples were fixed in 4% paraformaldehyde, embedded in paraffin, sectioned, and stained with hematoxylin and eosin (H&E). Histopathological evaluation was performed using light microscopy.

##### Gut Microbiome Metagenomics Analysis

Genomic DNA was extracted from rat fecal samples using the FastPure Stool DNA Isolation Kit (Magnetic Bead) (MJYH, Shanghai, China). Following extraction, DNA concentration and purity were measured, and integrity was assessed by 1% agarose gel electrophoresis. Fragmentation was performed using a Covaris M220 (Covaris, LLC, Woburn, MA, USA; distributed by Gene Company Limited, Shanghai, China), and fragments of approximately 350 bp were selected for paired-end (PE) library construction. Metagenomic sequencing was performed on an Illumina NovaSeq™ X Plus platform (Shanghai Meiji Biotechnology Co., Ltd., Shanghai, China).

Bioinformatics analysis was performed on the Majorbio Cloud Platform (https://cloud.majorbio.com). Quality control was conducted on the raw sequencing data, followed by assembly of high-quality reads to generate a non-redundant gene catalog. Following taxonomic annotation, α-diversity indices were calculated using R software (version 3.3.1). Principal coordinate analysis (PCoA) based on the Bray–Curtis distance matrix was performed to visualize β-diversity across samples. Relative taxonomic abundance at the phylum, genus, and species levels was determined by aligning the non-redundant gene catalog to the NR database. To identify inter-group microbial differences, multi-group comparative analysis and Linear Discriminant Analysis Effect Size (LEfSe) were employed. Differentially abundant gut microbial species were identified using thresholds of LDA score > 3 and *p* < 0.05. Spearman’s correlation analysis was used to examine the relationships between significantly enriched gut microbiota and relevant host indicators. Finally, functional metabolic pathways were annotated and subjected to enrichment analysis using the KEGG database.

##### Determination of SCFAs in Feces by Gas Chromatography

Fecal samples (0.1 g) were cryogenically homogenized in liquid nitrogen and the resulting powder was immediately transferred to 1.5 mL microcentrifuge tubes. An internal standard (2-ethylbutyric acid, final concentration: 2 mmol/L) and 0.4 mL of methanol were added, followed by thorough vortex mixing. The mixture was acidified to pH1 with HCl and incubated at room temperature for 10 min. After centrifugation at 13,776× *g* for 15 min, the supernatant was filtered through a 0.22 μm organic solvent-compatible membrane filter. Processed samples were analyzed immediately.

### 4.4. Statistical Analysis

Statistical analyses were performed using SPSS version 26.0. Continuous data are expressed as the mean ± standard deviation (mean ± SD). For comparisons between two groups, if data met assumptions of normality (assessed by the Shapiro–Wilk test) and homogeneity of variance (assessed by Levene’s test), the unpaired Student’s *t*-test was applied; otherwise, the nonparametric Mann–Whitney U test was used. For comparisons among more than two groups, one-way analysis of variance (ANOVA) was employed for normally distributed data with equal variances, followed by the least significant difference (LSD) post hoc test for multiple.

## 5. Conclusions

This study demonstrates that the seven probiotic strains obtained from equine colostrum possess favorable probiotic attributes and a sound safety profile, with significant cholesterol-lowering efficacy confirmed in both in vitro and in vivo models. The underlying mechanism involves the modulation of gut microbiota composition, function, and metabolic activity, which consequently ameliorates HFD-induced dyslipidemia and related metabolic dysregulation. Collectively, these findings establish a theoretical and experimental foundation for the potential application of these probiotics in the prevention and management of metabolic disorders, as well as in the development of associated functional food formulations.

## Figures and Tables

**Table 1:** Cholesterol degradation rate(%).

Strain Number	Nan1A_1_	Nan1A_2_	Nan1A_3_	Nan1B	Nan1C	J Nan1A	J Nan1B	Low-Dose	High-Dose
Cholesterol degradation rate/%	76.14 ± 0.77	86.29 ± 1.01	77.83 ± 1.59	83.11 ± 1.04	87.14 ± 0.83	45.07 ± 0.90	67.36 ± 0.99	50.52 ± 0.79	81.71 ± 1.06

**Table 2:** Effects of Composite Probiotics on Short-Chain Fatty Acid and Bile Acid Profiles in Hyperlipidemic Rats. The study included a sample size of 8 rats, with results expressed as mean ± standard deviation (SD).

Group	Acetate (mmol/L)	Propionate (mmol/L)	Butyrate (mmol/L)	Concentration (mmol/L)	TBA in Serum (μmol/L)	TBA in Fecal (μmol/L)
N	13.25 ± 1.05	1.98 ± 0.25	1.70 ± 0.24	16.92 ± 1.21	9.87 ± 1.13	8.07 ± 0.84
M	5.23 ± 1.29 **	1.04 ± 0.37 **	0.45 ± 0.14 **	6.71 ± 1.73 **	16.41 ± 1.47 **	15.30 ± 1.46 **
PC	8.50 ± 1.47 ^##^	1.79 ± 0.21 ^##^	1.69 ± 0.58 ^##^	11.99 ± 1.77 ^##^	9.87 ± 1.43 ^##^	8.60 ± 0.98 ^##^
Nan-D	6.13 ± 1.04	0.90 ± 0.15	0.53 ± 0.09	7.56 ± 1.19	15.26 ± 0.75	13.51 ± 1.03 ^##^
Nan-G	8.36 ± 1.19 ^##^	1.26 ± 0.33	0.80 ± 0.17 ^##^	10.42 ± 1.58 ^##^	10.80 ± 1.88 ^##^	10.11 ± 1.41 ^##^

** *p* < 0.001 vs. N, ^##^ *p* < 0.01 vs. M. These statistical comparisons highlight the significant differences between the groups in the study.

## Data Availability

Data is contained within the article. Further inquiries can be directed to the corresponding authors.

## Supplementary Materials

The following supporting information can be downloaded at https://www.mdpi.com/article/10.3390/ijms27177816/s1.

## References

[1] Sadowska A., Osiński P., Roztocka A., Chojnacka K.K., Zapora E., Sawicka D., Car H. (2023). Statins—From Fungi to Pharmacy. Int. J. Mol. Sci..

[2] Teng Y., Wang Y., Tian Y., Chen Y.-y., Guan W.-y., Piao C.-h., Wang Y.-h. (2020). Lactobacillus plantarum LP104 ameliorates hyperlipidemia induced by AMPK pathways in C57BL/6N mice fed high-fat diet. J. Funct. Foods.

[3] Yeerjiang B., Manaer T., Liu X., Bieerdimulati R., Nabi X. (2026). Mechanistic Insights into Lactobacillus harbinensis and Other Probiotics Regulating Lipid Metabolism in T2DM Mice via the PPARγ-LXRα-NPC1L1 Signaling Pathway Based on Multi-Omics Analysis. Metabolites.

[4] Albillos A., de Gottardi A., Rescigno M. (2020). The gut-liver axis in liver disease: Pathophysiological basis for therapy. J. Hepatol..

[5] Wu H., Chen J., Guo S., Deng J., Zhou Z., Zhang X., Qi T., Yu F., Yang Q. (2025). Advances in the acting mechanism and treatment of gut microbiota in metabolic dysfunction-associated steatotic liver disease. Gut Microbes.

[6] Martuzzi F., Franceschi P., Formaggioni P. (2024). Fermented Mare Milk and Its Microorganisms for Human Consumption and Health. Foods.

[7] Çetin B., Aktaş H. (2024). Monitoring probiotic properties and safety evaluation of antilisterial Enterococcus faecium strains with cholesterol-lowering potential from raw Cow’s milk. Food Biosci..

[8] Guo Q., Dong Q., Xu W., Zhang H., Zhao X., He W., He Y., Zhao G. (2024). Metabolite profiling of camel milk and the fermentation bacteria agent TR1 fermented two types of sour camel milk using LC-MS in relation to their probiotic potentials. Heliyon.

[9] Yu L., Manaer T., Xinhua N. (2015). Interactions of lactic acid bacterium and yeasts isolated from Xinjiang traditional fermented dairy products. Sci. Technol. Food Ind..

[10] Zhao X., Xie K., Wang Y., He Y., Wei H., Tao X. (2025). Screening of probiotic strains from human breast milk and evaluation of their potential anti-obesity properties. J. Funct. Foods.

[11] Bhushan B., Sakhare S.M., Narayan K.S., Kumari M., Mishra V., Dicks L.M.T. (2020). Characterization of Riboflavin-Producing Strains of Lactobacillus plantarum as Potential Probiotic Candidate through in vitro Assessment and Principal Component Analysis. Probiotics Antimicrob. Proteins.

[12] Zhang W., Lai S., Zhou Z., Yang J., Liu H., Zhong Z., Fu H., Ren Z., Shen L., Cao S.Z. (2022). Screening and evaluation of lactic acid bacteria with probiotic potential from local Holstein raw milk. Front. Microbiol..

[13] Song Y., Li A., Xu S., Ma L., Zhu H. (2022). Isolation, Screening, and Probiotic Function Evaluation of Lactic Acid Bacteria with Isolation, Screening, and Probiotic Function Evaluation of Lactic Acid Bacteria with. Mod. Food Sci. Technol..

[14] Campedelli I., Mathur H., Salvetti E., Clarke S., Rea M.C., Torriani S., Ross R.P., Hill C., O’Toole P.W. (2019). Genus-Wide Assessment of Antibiotic Resistance in *Lactobacillus* spp.. Appl. Environ. Microbiol..

[15] Ali S., Rasool S., Vajihe A. (2023). Probiotic Lactobacillus and the potential risk of spreading antibiotic resistance: A systematic review. Res. Pharm. Sci..

[16] Hummel A.S., Hertel C., Holzapfel W.H., Franz C.M. (2007). Antibiotic resistances of starter and probiotic strains of lactic acid bacteria. Appl. Environ. Microbiol..

[17] Agolino G., Pino A., Vaccalluzzo A., Cristofolini M., Solieri L., Caggia C., Randazzo C.L. (2024). Bile salt hydrolase: The complexity behind its mechanism in relation to lowering-cholesterol lactobacilli probiotics. J. Funct. Foods.

[18] MaLiNa K., Bao Y. (2021). Screening, Identification of Cholesterol-lowering Abilities Screening, Identification of Cholesterol-lowering Abilities Screening, Identification of Cholesterol-lowering Abilities. J. Chin. Inst. Food Sci. Technol..

[19] Ramasamy K., Abdullah N., Wong M., Karuthan C., Ho Y.W. (2010). Bile salt deconjugation and cholesterol removal from media by Lactobacillus strains used as probiotics in chickens. J. Sci. Food Agric..

[20] Kudaibergenova A., Begdildayeva N., Amirkhanova A., Nurgazina A., Ospanova A., Kondybayev A., Akhmetsadykova S., Jumagaziyeva A., Iskakbayeva Z., Jumabayeva S. (2025). Probiotic Characterization of Lactic Acid Bacteria Isolated from Traditional Fermented Camel Milk and Horse Milk Products. ES Food Agrofor..

[21] Sonnenburg J.L., Sonnenburg E.D. (2019). Vulnerability of the industrialized microbiota. Science.

[22] Zhu Q., Xing G., Shan Z., Luo S., Mao Y., Ren L., Wang T., Ma Y., Liu Y., Liu J. (2025). Screening and Genome Analysis of Potential Probiotic *Lactiplantibacillus plantarum* with Anti-Listeria monocytogenes Activity from Traditional Fermented Foods. Microorganisms.

[23] Kanimozhi R., Sangavi R., Malligarjunan N., Gowrishankar S. (2025). Screening, isolation, identification and evaluation of bacteria with probiotic potential from traditional palmyra palm nectar. Front. Cell. Infect. Microbiol..

[24] Gao Y., Huang F., Wang J., Wang H., Kong B., Qin L., Chen Q. (2023). Research Progress on Cholesterol-Lowering Mechanism and Evaluation Strategies for Probiotics. Food Sci..

[25] Qu T., Yang L., Wang Y., Jiang B., Shen M., Ren D. (2020). Reduction of serum cholesterol and its mechanism by Lactobacillus plantarum H6 screened from local fermented food products. Food Funct..

[26] Kenny D.J., Plichta D.R., Shungin D., Koppel N., Hall A.B., Fu B., Vasan R.S., Shaw S.Y., Vlamakis H., Balskus E.P. (2020). Cholesterol Metabolism by Uncultured Human Gut Bacteria Influences Host Cholesterol Level. Cell Host Microbe.

[27] Wen J.-J., Li M.-Z., Gao H., Hu J.-L., Nie Q.-X., Chen H.-H., Zhang Y.-L., Xie M.-Y., Nie S.-P. (2021). Polysaccharides from fermented *Momordica charantia* L. with Lactobacillus plantarum NCU116 ameliorate metabolic disorders and gut microbiota change in obese rats. Food Funct..

[28] Louis P., Young P., Holtrop G., Flint H.J. (2010). Diversity of human colonic butyrate-producing bacteria revealed by analysis of the butyryl-CoA: Acetate CoA-transferase gene. Environ. Microbiol..

[29] Melanie L.S., Yifan X., Manolo L., Alvarez G.F., Daphne K., Chandler D., Uku K., Sarah A., Manon L., Pascale F. (2022). The effects of Aronia berry (poly)phenol supplementation on arterial function and the gut microbiome in middle aged men and women: Results from a randomized controlled trial. Clin. Nutr..

[30] Li Y., Chen M., Ma Y., Yang Y., Cheng Y., Ma H., Ren D., Chen P. (2022). Regulation of viable/inactivated/lysed probiotic Lactobacillus plantarum H6 on intestinal microbiota and metabolites in hypercholesterolemic mice. npj Sci. Food.

[31] Chen Y., Deng Q., Xu J., Wang X., Hu C., Tang H., Huang F. (2018). Sinapic acid and resveratrol alleviate oxidative stress with modulation of gut microbiota in high-fat diet-fed rats. Food Res. Int..

[32] Wei X., Tao J., Xiao S., Jiang S., Shang E., Zhu Z., Qian D., Duan J. (2018). Xiexin Tang improves the symptom of type 2 diabetic rats by modulation of the gut microbiota. Sci. Rep..

[33] Wang G., Xia C., Li W., Lin Z., Yong X., Lian A. (2020). Diverse conditions contribute to the cholesterol-lowering ability of different Lactobacillus plantarum strains. Food Funct..

[34] Li H., Liu F., Lu J., Shi J., Guan J., Yan F., Li B., Huo G. (2020). Probiotic Mixture of Lactobacillus plantarum Strains Improves Lipid Metabolism and Gut Microbiota Structure in High Fat Diet-Fed Mice. Front. Microbiol..

[35] Collins S.L., Stine J.G., Bisanz J.E., Okafor C.D., Patterson A.D. (2022). Bile acids and the gut microbiota: Metabolic interactions and impacts on disease. Nat. Rev. Microbiol..

[36] Na R., Ru Y., Liu W., MengHe B. (2023). Isolation and identification of lactic acid bacteria in koumiss in different regions Central and East Asia and screening of excellent strains. Food Ferment. Ind..

[37] Xiao C., Yue X., Wu X., Ye Z., Yang F. (2025). In Vitro Probiotic Properties and Antioxidant Activity of Lactobacillus945 crispatus DY38 from Goats. Chin. J. Anim. Nutr..

[38] Chen D., Cheng Y., Ren C., Chen C., Qu H., Wa Y., Yan X., Chen X., Huang Y., Gu R. (2022). Capability of Lactobacillus to Tolerate Digestive Stress and Effect of Digestive Stress on Its Intestinal Adhesion Capacity. Food Sci..

[39] Samedi L., Charles A.L. (2019). Isolation and characterization of potential probiotic Lactobacilli from leaves of food plants for possible additives in pellet feeding. Ann. Agric. Sci..

[40] Yang Q., Wang R., Sun Y., Liu X., Li Q., Ma Y. (2023). Screening, identification and properties of cholesterol lowering lactic acid bacteria. Food Ferment. Ind..

