# Supplementary material for: Multiple Probiotic Lactic Acid Bacteria with Marked Cholesterol-Lowering Effects Were Discovered in Equine Colostrum
Source: Int J Mol Sci. 2026 Aug 31;27(17):7816. doi: 10.3390/ijms27177816 (PMC13566805; doi:10.3390/ijms27177816)
Supplement: Supplementary file 1 [file ijms-27-07816-s001.zip › ijms-4487712-supplementary.pdf]

Supplementary material

Table S1. Morphological Characteristics of Lactic Acid Bacteria and Yeasts

Strain	Size	Texture	Elevation	Margin	Transparency	Smoothness	Luster	Color
Nan1A_1	Large	Moist	Convex	Entire	Opaque	Smooth	Present	White
Nan1A_2	Medium	Moist	Convex	Entire	Opaque	Smooth	Present	Creamy white
Nan1A_3	Small	Moist	Convex	Entire	Opaque	Smooth	Present	White
Nan1B	Small	Moist	Slightly convex	Entire	Opaque	Smooth	Present	Grayish white
Nan1C	Small	Moist	Convex	Entire	Opaque	Smooth	Present	Creamy white
J Nan1A	Large	Viscous	Flat	Irregular	Opaque	Rough	Absent	White
J Nan1B	Large	Viscous	Flat	Irregular	Opaque	Rough	Absent	White

Table S2. Physiological and Biochemical Characteristics of Lactic Acid Bacteria

	Nan1A_1	Nan 1A_2	Nan 1A_3	Nan 1B	Nan 1C		Nan 1A_1	Nan 1A_2	Nan 1A_3	Nan 1B	Nan 1C
Cell Shape	Rod	Rod	Rod	Rod	Rod	α-Methyl-D-glucoside	-	-	-	-	-
Gram Staining	+	+	+	+	+	N-Acetylglucosamine	+	+	+	+	+
Catalase	-	-	-	-	-	Amygdalin	+	+	+	+	+
Oxidase	-	-	-	-	-	Arbutin	+	+	+	+	+
Indole Test	-	-	-	-	-	Esculin	+	+	+	+	+
Gelatin Liquefaction	-	-	-	-	-	Salicin	+	+	+	+	+
Growth at 45°C	-	+	+	-	-	Cellobiose	+	+	+	+	+
Growth in 6.5% NaCl	-	-	-	-	-	Maltose	+	+	+	+	+
Glycerol	-	-	-	-	-	Lactose	+	+	+	+	+
Erythritol	-	-	-	-	-	Melibiose	+	+	+	+	+
D-Arabinose	-	-	-	-	-	Sucrose	+	+	+	+	+
L-Arabinose	-	+	+	+	+	Trehalose	+	+	+	+	+
D-Ribose	-	+	+	+	+	Inulin	-	-	-	-	-
D-Xylose	-	-	-	-	-	Melezitose	-	+	+	+	+
L-Xylose	-	-	-	-	-	Raffinose	+	+	+	+	+
Adonitol	-	-	-	-	-	Starch	+	-	-	-	-
β-Methyl-D-xyloside	-	-	-	-	-	Glycogen	-	-	-	-	-
D-Galactose	+	+	+	+	+	Xylitol	-	-	-	-	-
D-Glucose	+	+	+	+	+	Gentiobiose	+	+	+	+	+
D-Fructose	+	+	+	+	+	D-Turanose	-	-	-	-	-
D-Mannose	-	+	+	+	+	D-Lyxose	-	-	-	-	-
L-Sorbose	-	-	-	-	-	D-Tagatose	-	-	-	-	-
L-Rhamnose	-	-	-	-	-	*D*-Fucose	-	-	-	-	-
Dulcitol	-	-	-	-	-	L-Fucose	-	-	-	-	-
Inositol	-	-	-	-	-	D-Arabitol	-	-	-	-	-
Mannitol	-	-	+	+	+	L-Arabitol	-	-	-	-	-
Sorbitol	-	+	+	+	+	Gluconate	-	-	-	-	-
α-Methyl-D-mannoside	-	+	+	+	+	2-Ketogluconate	-	-	-	-	-

Note: "+" indicates positive result; "-" indicates negative result.

Table S3. Sugar Fermentation and Growth Characteristics of Yeast under Various Conditions

	**JNan1A**	**JNan1B**		**J Nan1A**	**J Nan1B**
Cell Shape	Ellipsoidal	Ellipsoidal	Growth at 37°C	+	-
Corn Starch Agar (Dalmau Medium)	+	+	Growth at 40°C	-	-
Malt extract liquid medium	+	+	Malt Extract Agar Medium	+	+
Glucose	+	-	Maltose	-	-
Galactose	-	-	Lactose	-	-
Sucrose	-	-	Raffinose	-	-

Note: "+" indicates positive result; "-" indicates negative result.

Table S4. Carbon Source Assimilation Characteristics of Yeast

	J Nan1A	J Nan1B		J Nan1A	J Nan1B
Glucose	+	+	L-Rhamnose	-	-
Galactose	-	-	Methanol	-	-
L-Sorbose	-	-	Ethanol	+	+
Sucrose	-	-	Glycerol	-	+
Maltose	-	-	Erythritol	-	-
Cellobiose	-	-	Ribitol	-	-
Trehalose	-	-	Galactitol	-	-
Lactose	-	-	D-Mannitol	-	-
Melibiose	-	-	D-Sorbitol	-	-
Raffinose	-	-	Methyl Glucoside	-	-
Melezitose	-	-	Salicin	-	-
Soluble Starch	-	-	Lactic Acid	+	+
D-Xylose	+W	-	Succinic Acid	+	+
L-Arabinose	-	-	Citric Acid	+	-
D-Ribose	-	-	Inositol	-	-

Note: "+" indicates positive result; "-" indicates negative result; "+W" indicates weak positive result

Table S5. Nitrogen Source Assimilation Characteristics of Yeast

	J Nan1A	J Nan1B		J Nan1A	J Nan1B
Nitrate	-	-	Cadaverine Dihydrochlo	-	-
Sarcosine	-	-	Lysine	-	-

Note: "+" indicates positive result; "-" indicates negative result

Table S6. Types of drug-sensitive paper tablets, drug content and determination criteria

Antibiotic susceptibility test discs Types	Paper strip potency (μg)	Judgment Criteria (Inhibition Zone Diameter / mm)		
		Drug Resistance (R)	Intermediate (I)	Susceptible (S)
Amoxicillin (AMX)	10	≤13	14~17	≥18
Cefamandole(MAN)	30	≤14	15~17	≥18
Ceftazidime(CAZ)	30	≤14	15~17	≥18
Gentamicin(GEN)	10	≤12	13~14	≥15
Azithromycin (AZM)	15	≤13	14~17	≥18
Doxycycline(DOX)	30	≤12	13~15	≥16
Vancomycin(VAN)	30	≤14	15~6	≥17
Ciprofloxacin(CIP)	5	≤15	16~20	≥21
Amphotericin B (AMB)	10	≤10	11~14	≥15
Fluconazole (FLU)	25	≤11	12~19	≥20
Itraconazole (ITC)	8	≤9	10~14	≥15
5-fluorocytosine (5-FC)	1	≤22	23~29	≥30
